# The Trends in Global Gene Expression in Mouse Embryonic Stem Cells During Spaceflight

**DOI:** 10.3389/fgene.2019.00768

**Published:** 2019-09-06

**Authors:** Lili An, Yanming Li, Yingjun Fan, Ning He, Fanlei Ran, Hongzhu Qu, Yanqiu Wang, Xuetong Zhao, Chen Ye, Yuanda Jiang, Xiangdong Fang, Haiying Hang

**Affiliations:** ^1^Key Laboratory of Protein and Peptide Drugs, National Laboratory of Biomacromolecules, Institute of Biophysics, Chinese Academy of Sciences, Beijing, China; ^2^CAS Key Laboratory of Genome Sciences and Information, Beijing Institute of Genomics, Chinese Academy of Sciences, Beijing, China; ^3^Institute for Stem Cell and Regeneration, Chinese Academy of Sciences, Beijing, China; ^4^College of Life Sciences, University of Chinese Academy of Sciences, Beijing, China; ^5^Sino-Danish College, University of Chinese Academy of Sciences, Beijing, China; ^6^Center for Space Science and Applied Research, Chinese Academy of Sciences, Beijing, China

**Keywords:** spaceflight, Rad9, mouse embryonic stem cells, gene expression profile, genomic DNA lesions

## Abstract

The environment in space differs greatly from the environment on the ground. Spaceflight causes a number of physiological changes in astronauts, such as bone loss and immune system dysregulation. These effects threaten astronauts’ space missions, and understanding the underlying cellular and molecular mechanisms is important to manage the risks of space missions. The biological effects of spaceflight on mammalian cells, especially with regards to DNA damage, have attracted much attention. *Rad9* ^−/−^ mouse embryonic stem cells (mESCs) are known to be extremely sensitive to DNA damage agents. In this study, a project of the SJ-10 satellite programme, we investigated the gene expression profiles of both *Rad9* ^−/−^ mESCs and *Rad9* ^+/+^ (wild-type) mESCs in space with a focus on genes critical for inducing, preventing, or repairing genomic DNA lesions. We found that spaceflight downregulated more genes than it upregulated in both wild-type and *Rad9* ^−/−^ mESCs, indicating a suppressive effect of spaceflight on global gene expression. In contrast, *Rad9* deletion upregulated more genes than it downregulated. Of note, spaceflight mainly affected organ development and influenced a wide range of cellular functions in mESCs, while *Rad9* deletion mainly affected the development and function of the hematological system, especially the development, differentiation and function of immune cells. The patterns of gene expression in mouse embryonic stem cells in space is distinct from those in other types of cells. In addition, both spaceflight and *Rad9* deletion downregulated DNA repair genes, suggesting a possibility that spaceflight has negative effects on genome for embryonic stem cells and the effects are likely worsen when the genome maintenance mechanism is defective.

## Introduction

Spaceflight causes a number of changes in astronauts, such as bone loss, skeletal muscle atrophy, cardiovascular problems, and immune system dysregulation (White and Averner, 2001; Hatton et al., 2002; Allen et al., 2009; Brian and Clarence, 2009) as well as changes in cellular morphology, locomotion, trans-membrane signalling, metabolism, and cell–cell association (Harris et al., 2000; Kunz et al., 2017). The effects of spaceflight on gene expression profiles have been studied in various mammalian cells, such as human umbilical vein endothelial cells (Versari et al., 2013), human renal cortical cells (Hammond et al., 2000), human fibroblast cells (Liu and Wang, 2008; Zhang et al., 2016), and osteogenic murine bone marrow stromal cells (Monticone et al., 2010). To our knowledge, however, there has been no such report on mouse embryonic stem cells (mESCs), which we investigated in this study.

The integrity of genomic DNA is vital for the normal physiological functions of cells (Lombard et al., 2005). Cells have evolved an intricate system to rectify DNA errors and damage; the genome-protecting system is composed of many proteins, including cell cycle checkpoint proteins and DNA repair factors. Rad9 plays important roles in both cell cycle checkpoint control and DNA repair (Hartwell and Weinert, 1989; Paulovich and Hartwell, 1995; Hopkins et al., 2004; Lieberman, 2010). *Rad9* ^−/−^ mESCs demonstrate remarkable increases in spontaneous chromosomal aberrations and hypoxanthine–guanine phosphoribosyltransferase (HPRT) mutations and are extremely sensitive to DNA damage agents such as gamma rays, UV light, and hydroxyurea relative to wild-type mESCs (Hopkins et al., 2004). We have also found that *Rad9* deletion reduces the expression of nucleotide excision repair factor genes (Li et al., 2013). In addition, we have demonstrated that *Rad9* deletion enhances the activity of superoxide dismutase and catalase (two antioxidant factors) but has no effect on the gene expression of NOX2, a major free radical-generating factor (Li et al., 2015). However, we have not systematically examined how *Rad9* deletion affects the gene expression of DNA repair factors, free radical generating factors and antioxidants on a genome scale. These three types of proteins are major factors involved in counteracting/inducing DNA damage.

Because of the existence of space radiation, the effects of spaceflight on DNA damage have attracted much attention. Researchers using fixed, frozen, or cultured mammalian cells as models have detected double strand breaks (DSBs) induced by spaceflight (Dieriks et al., 2009; Ohnishi et al., 2009; Yatagai et al., 2011; Lu et al., 2017). Furthermore, increased occurrence of chromosomal aberrations has been observed in the blood cells of astronauts after long missions (George et al., 2001). However, because of the high costs of and limited access to spaceflight, the low doses and dose rates of space radiation, and the involvement of other environmental factors in space such as microgravity, our knowledge of the effects of space exposure on DNA damage is limited (Moreno-Villanueva et al., 2017). Thus, more studies using various cell models in the space environment are needed. As mentioned above, *Rad9* ^−/−^ mESCs are extremely sensitive to DNA damage agents. Thus, wild-type and *Rad9* ^−/−^ mESCs are ideal models with which to investigate the effects of spaceflight on DNA damage and its interaction with the genome maintenance system.

We designed experiments to investigate the effects of spaceflight on global gene expression in mESCs. One objective of our research was to study how spaceflight influences the expression of genes critical for inducing, preventing or repairing genomic DNA lesions in mouse mESCs. Recoverable satellites are very useful tools for space experiments. The 24^th^ recoverable satellite of China was designed specifically for experiments on microgravity physics and space life science that are collectively referred to as the SJ-10 programme (Hu et al., 2014). As one of the projects in the recovery satellite SJ-10 programme, we investigated the gene expression profiles of both *Rad9* ^−/−^ mESCs and wild-type mESCs after 1 day or 5 days of culture in space. We found that spaceflight had a suppressive effect on global gene expression in mESCs. Furthermore, spaceflight-induced changes in global gene expression were weakened by *Rad9* deletion.

## Materials and Methods

### Sample Preparation

This experiment was performed in the payload onboard the SJ-10 satellite. This payload was designed and prepared by the National Center of Space Science, Chinese Academy of Sciences, and is mainly composed of four mouse cell culture devices, four fruit fly culture containers, three temperature controllers, two fluid reservoirs, one discharge liquid reservoir, two injection pumps, a liquid distributor, an electronic driver controller, and a three-segment air-proof payload body. Two fluid reservoirs were used for storage of RNAlater (Ambion, Shanghai, China), and one discharge liquid reservoir was used for storage of the waste liquids.

**Figure 1:**
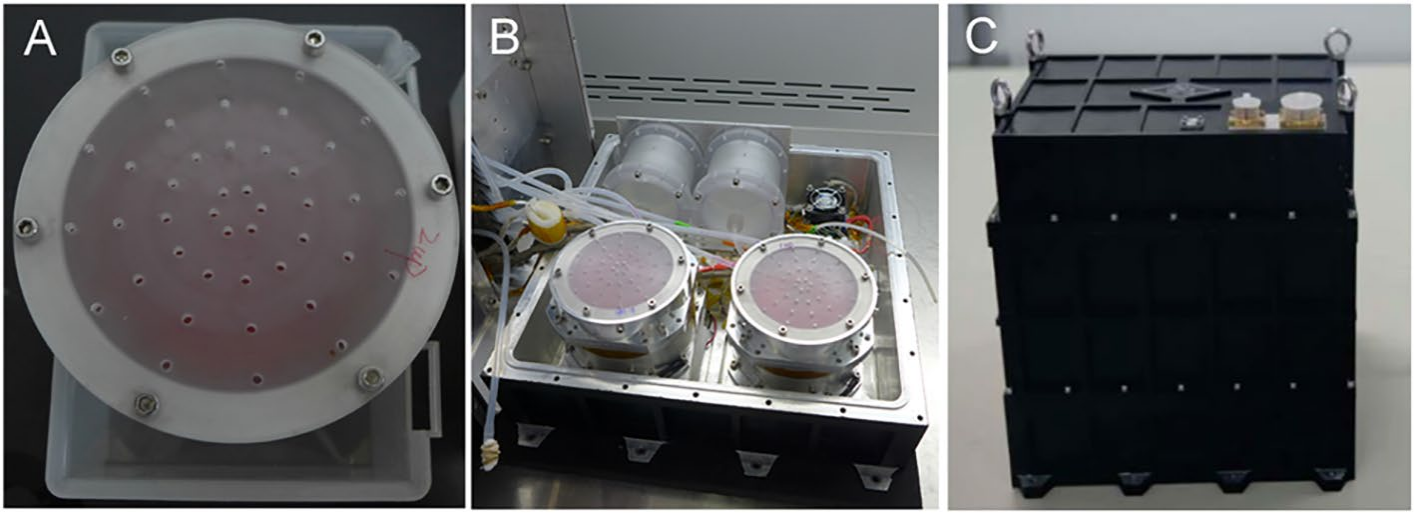
Cell culture devices and payload. **(A)** Assembled cell culture device containing cells and medium. **(B)** All four cell culture devices were installed in the lower part of the three-segment payload. **(C)** Sealed payload.

The cell culture device was mainly composed of a cell culture plate, an upper cover, a demarcation plate, a rolling film and a hollow connector. First, the cells were seeded on the culture plate, which was placed in a 10 cm culture dish and cultured in a CO_2_ incubator. After adhesion, the culture plates were assembled into the cell culture devices under aseptic conditions. Then, 50 ml of medium was injected into each cell culture device without introducing bubbles and the cell culture devices were assembled into the payload before the launch of the satellite. Images of the cell culture devices before and after their installation into the payload are shown in **Figure 1**.

There was no O_2_ and CO_2_ controller for the culture devices. We took the following measures to ensure the culture of the mESCs without an O_2_ and CO_2_ controller. 1) We injected 50 ml of fresh and 5% CO_2_-balanced medium into the cell culture devices. 2) The cell culture device was gas-permissible, while the payload was airtight. Before the satellite launch, the whole payload was placed in a CO_2_ incubator with 95% air/5% CO_2_ for 30 min, then air-tightly sealed as soon as possible. In our simulating experiments performed on the ground, we confirmed that the mESCs grew well in the payload without an O_2_ and CO_2_ controller, and the pH of the medium from the cell culture devices after 5 days, culture was in the normal range.

The purpose of our study is to investigate the gene expression profiles of both *Rad9* ^−/−^ mESCs and wild-type mESCs after 1 day or 5 days of culture in space. Therefore, we have four cell culture devices in the payload, which designated cell culture device A (wild-type mESCs of 1 day culture in space), B (wild-type mESCs of 5 days culture in space), C (*Rad9* ^−/−^ mESCs of 1 day culture in space) and D (*Rad9* ^−/−^ mESCs of 5 days culture in space). The numbers of the cells seeded in each cell culture device were the key point of this study which were determined by the preliminary experiments on the ground. That is, for both *Rad9* ^+/+^ and *Rad9* ^−/−^ mESCs, seeding of 10^6^ cells for 1 day of culture was suitable. For *Rad9* ^+/+^ mESCs, seeding of 2 × 10^5^ cells for 5 days of culture was suitable. Compared to *Rad9* ^+/+^ mESCs, *Rad9* ^−/−^ mESCs grows relatively slower, and 4 × 10^5^ cells for 5 days of culture was suitable.

Cell detachment was another point we should concern in this study. The medium was injected into the space between the cell culture plate and the rolling film. The rolling film was flexible and the medium was wrapped by the rolling film. The demarcation plate was placed between the cell culture plate and the rolling film to protect the cells from detachment because of the movement of the rolling film. In our previous experiments using the 3D-clinostat on the ground, we observed few cell detachments. In the current study, although we were not able to judge the accurate percentage of detached cells during the flight, many cells were still attached on the cell culture plate after the return of the satellite.

**Figure 2:**
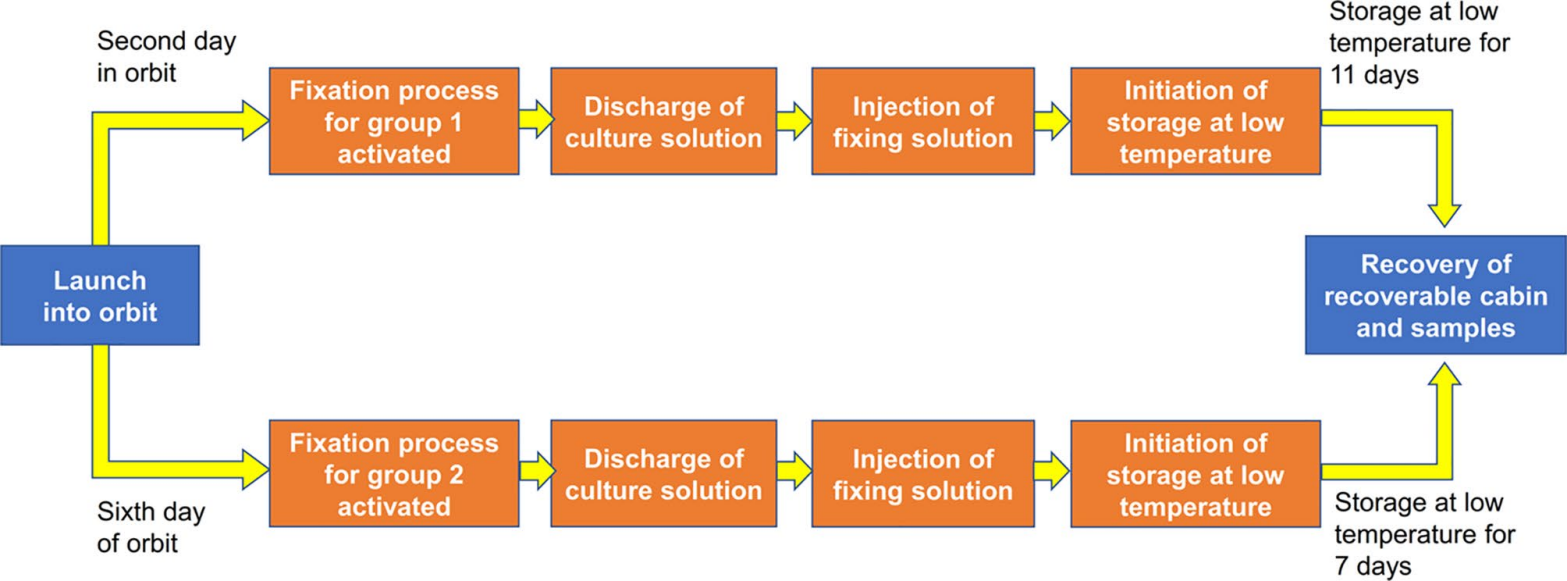
Flow chart of the experiment. We seeded 1 × 10^6^ wild-type mESCs for 1 day of culture (cell culture device A), 2 × 10^5^ wild-type mESCs for 5 days of culture (cell culture device B), 1 × 10^6^*mRad9*^-/-^ mESCs for 1 day of culture (cell culture device C) and 4 × 10^5^ *mRad9* ^−/−^ mESCs for 5 days of culture (cell culture device D). The four cell culture devices were divided into two groups, with cell culture devices A and C in group 1 and cell culture devices B and D in group 2.

The four cell culture devices were divided into two groups; cell culture devices A and C were in group 1, and cell culture devices B and D were in group 2. There was a separate temperature control device under each group. The cells were cultured at 37 ± 0.5°C. The devices automatically changed liquids at predetermined time points with a change rate of 5 ml per 3 min. One day after the satellite was launched into orbit, the medium in cell culture devices A and C was discharged into the discharge liquid reservoir, and RNAlater was then injected into the cell culture devices to fix the cells. After fixation, the cells were stored in a low-temperature environment of 8 ± 2°C. Five days after the satellite was launched into orbit, the medium in cell culture devices B and D was discharged into the discharge liquid reservoir, and RNAlater was then injected into the cell culture devices to fix the cells. After fixation, the cells are stored in a low-temperature environment of 8 ± 2°C (**Figure 2**). After the return of the satellite, the culture plates were removed from the cell culture devices and washed with PBS. Then, the cells were lysed with TRIzol (Invitrogen) for RNA extraction and subsequent RNA-sequencing (RNA-seq) analysis. In our preliminary experiments on the ground, we have tested the reliability of each step. We confirmed that the quality of the RNA extracted from the samples fixed with RNAlater and stored at 8°C for 15 days was good enough for subsequent sequencing analysis. We also performed the vibration experiment to make sure that the samples in the payload can withstand extreme conditions during the launching and return of the satellite. The photos of the cells just before the assembly into the payload and the photos of the fixed cells after the return of the satellite were shown in **Figure S1**.

Identical procedures were performed on wild-type mESCs and *Rad9* ^−/−^ mESCs on the ground, which served as the controls. That is, both the samples of the ground group and the space group were cultured in the payload with the same procedure including the number of the cells seeded in each cell culture device, the work flow of the assembly of the payload, the setting of the temperature at the culture stage and the fixation stage of the sample and the processing of the fixed samples after their taking out from the cell culture device. The differences between the ground group and the space group were listed in **Table 1**.

**Table 1:** The differences between the ground group and the space group.

Culture conditions	Ground group	Space group
Microgravity	No	Yes
Space radiation	No	Yes
Other environmental stress in space	No	Yes
Vibration during the launch of the satellite	No	Yes
Vibration during the return of the satellite	No	Yes

### Sequencing

Total RNA from each cell sample was extracted by using TRIzol reagent following the manufacturer’s instructions. Libraries were constructed using Illumina mRNA-Seq library preparation kits. The concentration and size distribution of the libraries were determined by using an ABI 7500 instrument and an Agilent Bioanalyzer DNA 2000 chip (Agilent Technologies, Santa Clara, CA, USA), respectively. Qualified libraries were subjected to sequencing on an Illumina HiSeq 3000 Genome Analyzer platform with 2 × 100-bp paired-end reads.

### Data Processing and Analyses

Quality control analysis of FASTQ format raw sequencing data was performed using FastQC software. FASTQ format sequencing reads were mapped to the mm9 reference genome using TopHat version 2.0.9 software with the default parameters. The R package edgeR was used to identify differentially expressed genes (DEGs) with a threshold of fold change ≥ 2 and *p* < 0.05. Gene Ontology (GO) analysis and Ingenuity Pathways Analysis (IPA) were performed to identify the main enriched biological functions of the DEGs. The raw sequence data reported in this paper have been deposited in the following two repositories. One is the Genome Sequence Archive of Beijing Institute of Genomics, Chinese Academy of Sciences, under accession numbers CRA001384 that are publicly accessible at http://bigd.big.ac.cn/gsa. The other is the Gene Expression Omnibus (GEO), under accession numbers GSE134090 (https://www.ncbi.nlm.nih.gov/geo/query/acc.cgi?acc=GSE134090).

### Quantitative Real-Time PCR

Total RNA was extracted from each cell sample by using TRIzol reagent following the manufacturer’s instructions (Life Technologies). Reverse transcription was performed using the SuperScript^®^ III First-Strand Synthesis System for RT-PCR (Invitrogen). Real-time PCR was performed using the StepOnePlus^TM^ system (Life Technologies) with SYBR^®^ Green I (Takara) to label amplified DNA. Gene expression was normalized to that of the housekeeping gene *GAPDH*. Nine genes were randomly selected for quantitative real-time PCR to evaluate the expression data from RNA-seq. The primer sequences are listed in **Table 2**.

**Table 2:** Sequences of the primers used in quantitative real-time PCR analysis.

Target gene	Primer sequence (5′-3′)
Ube2s	GCAGACTCTGGGTTAGGGTG
	AATGGCGAGATCTGTGTCAA
Plac1	TCTTGCAGCAGGTTAGGTGA
	AAGCCACGTTTCAAAGGAGA
Lefty1	GGAGGTCTCTGACACCAGGA
	CTGCTACAACACAGCCATGC
Apoe	AGGCATCCTGTCAGCAATGT
	GGACTTGTTTCGGAAGGAGC
Fabp3	CTTGGTCATGCTAGCCACCT
	CTTTGTCGGTACCTGGAAGC
Neu1	GTGTCCACACACAATGAGCC
	CCCGGAATCTCTCTGTGGA
Myc	TGAAGTTCACGTTGAGGGG
	AGAGCTCCTCGAGCTGTTTG
Stac2	GGAGAGGGAGCGCTTAAATC
	TTATACCCCAACCATGACCG
Wnt8a	GGATGGCATGAATGAAGGAT
	GGTGGAATTGTCCTGAGCAT

## Results

### Gene Expression Profiles of Wild-Type and *Rad9* ^−/−^ mESCs in Spaceflight or on the Ground

**Figure 3:**
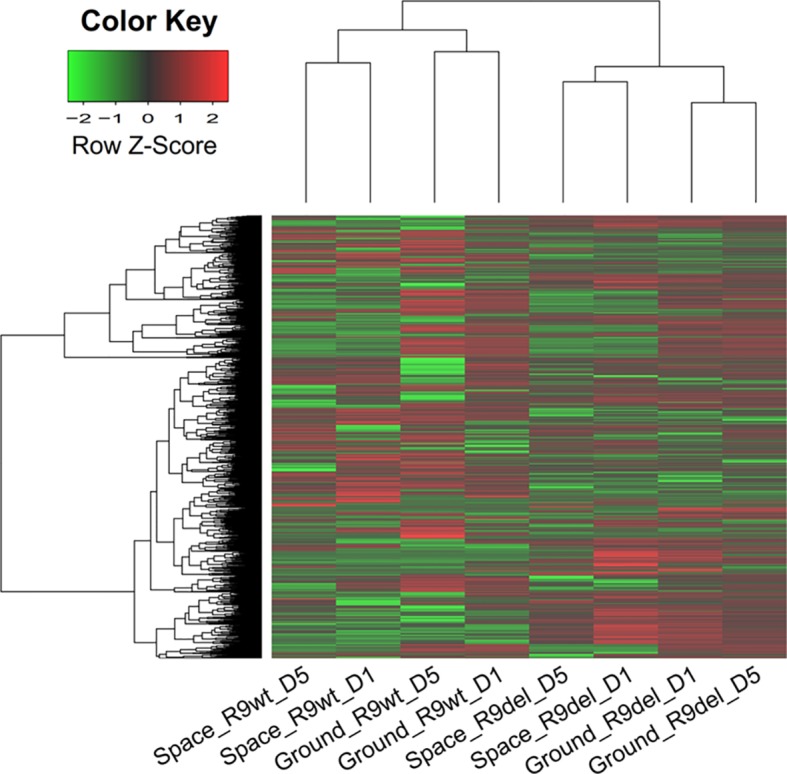
Hierarchical clustering of the expression of all DEGs (identified with a single-sample comparison strategy) in eight mESC samples. Each row represents a gene, while each column represents a sample. The expression intensities range from green (low expression) to red (high expression). The dendrograms at the top and left of the heat map suggest the similarities among the samples and genes, respectively. Space, mESCs cultured in space; Ground, mESCs cultured on the ground; R9wt, wild-type mESCs; R9del, *Rad9* *^−/−^* mESCs; D5, mESCs cultured for 5 days; D1, mESCs cultured for 1 day.

We analyzed 8 cell samples in total, which were designated as Space_R9wt_D1, Space_R9wt_D5, Space_R9del_D1, Space_R9del_D5, Ground_R9wt_D1, Ground _R9wt_D5, Ground _R9del_D1, and Ground_R9del_D5. These samples differed with respect to three variables: location (space or ground), genotype (R9wt or R9del, corresponding to wild-type or *Rad9*^-/-^ mESCs, respectively) and culture duration (D1 or D5, corresponding to 1 day or 5 days of culture, respectively). The culturing, RNA isolation and sequencing methods for these samples are described in detail in the *Materials and methods* section. After evaluating the quality of the sequencing data (**Figure S2**), we identified the DEGs using a threshold of fold change ≥ 2 and *p* < 0.05. Overall, we identified 5,122 DEGs (after eliminating repeats), which are displayed in a heat map according to their expression patterns (**Figure 3**). Each row represents one gene, while each column represents one cell sample type. Unsupervised hierarchical clustering indicated that these eight samples were clustered into two main groups, R9wt and R9del; each group was further clustered into two sub-groups, space and ground. These results indicate that both *Rad9* deletion and spaceflight lead to significant molecular alterations in mESCs, while culture duration has much less influence on mESCs.

To validate the RNA-seq results, we randomly selected nine genes and detected their expression in all eight samples using qRT-PCR analysis. For each gene, we compared the results from qRT-PCR and RNA-seq for all eight samples and calculated their correlation coefficients. As shown in **Figure S3A**, the PCR results closely reflected the RNA-seq results. For example, the *Neu1* expression levels derived from RNA-seq were closely correlated with those derived from qRT-PCR (R = 0.9583, **Figure S3B**). Thus, the RNA-seq data were considered reasonably reliable for further analyses.

By identifying DEGs between two samples that differed in only one variable (with the other two variables identical), we produced 12 sets of DEGs reflecting the alterations caused exclusively by spaceflight, *Rad9* gene deletion, or culture duration (**Table 3**). The first four sets of DEGs shown in **Table 3** (rows 1 to 4) reflect the effects of spaceflight on the gene expression profiles of mESCs. In comparisons of space samples with ground samples, 372 genes were upregulated and 674 genes were downregulated in wild-type mESCs cultured for 1 day, 710 genes were upregulated and 987 genes were downregulated in wild-type mESCs cultured for 5 days, 92 genes were upregulated and 311 genes were downregulated in *mRad9* ^−/−^ mESCs cultured for 1 day, and 91 genes were upregulated and 380 genes were downregulated in *mRad9* ^−/−^ mESCs cultured for 5 days. Of note, spaceflight led to more downregulation than upregulation, indicating a suppressive effect of spaceflight on global gene expression in mESCs.

**Table 3:** Number of DEGs between two samples differing in only one variable.

Compared conditions	Fixed conditions	Upregulated genes	Downregulated genes
Space/ground	R9wt D1	372	674
	R9wt D5	710	987
	R9del D1	92	311
	R9del D5	91	380
R9del/R9wt	Space D1	1,110	596
	Space D5	286	291
	Ground D1	527	190
	Ground D5	778	444
D5/D1	Space R9wt	458	288
	Space R9del	96	213
	Ground R9wt	476	571
	Ground R9del	32	31

Note: Space, mESCs cultured in space; Ground, mESCs cultured on the ground; R9wt, wild-type mESCs; R9del, Rad9*^−/−^* mESCs; D5, mESCs cultured for 5 days; D1, mESCs cultured for 1 day.

In comparisons of *mRad9* ^−/−^ mESCs with wild-type mESCs, 1,110 genes were upregulated and 596 genes were downregulated in the samples cultured for 1 day in space, 286 genes were upregulated and 291 genes were downregulated in the samples cultured for 5 days in space, 527 genes were upregulated and 190 genes were downregulated in the samples cultured for 1 day on the ground, and 778 genes were upregulated and 444 genes were downregulated in the samples cultured for 5 days on the ground. Thus, *Rad9* deletion led to more upregulation than downregulation (**Table 3**, rows 5 to 8).

Notably, we did not observe synergistic inhibitory effects of spaceflight and *Rad9* gene expression (contrary to its deletion) on global gene expression in mESCs. For example, *Rad9* deletion led to upregulation of 527 genes and downregulation of 190 genes in the cells cultured on the ground for 1 day. In cells experiencing spaceflight for 1 day, *Rad9* deletion led to upregulation of 1110 genes and downregulation of 596 genes. Thus, spaceflight not only increased the number of genes downregulated upon *Rad9* deletion but also increased the number of genes upregulated upon *Rad9* deletion.

### Overall Effects of Spaceflight and *Rad9* Gene Deletion on Gene Expression in mESCs

**Figure 4:**
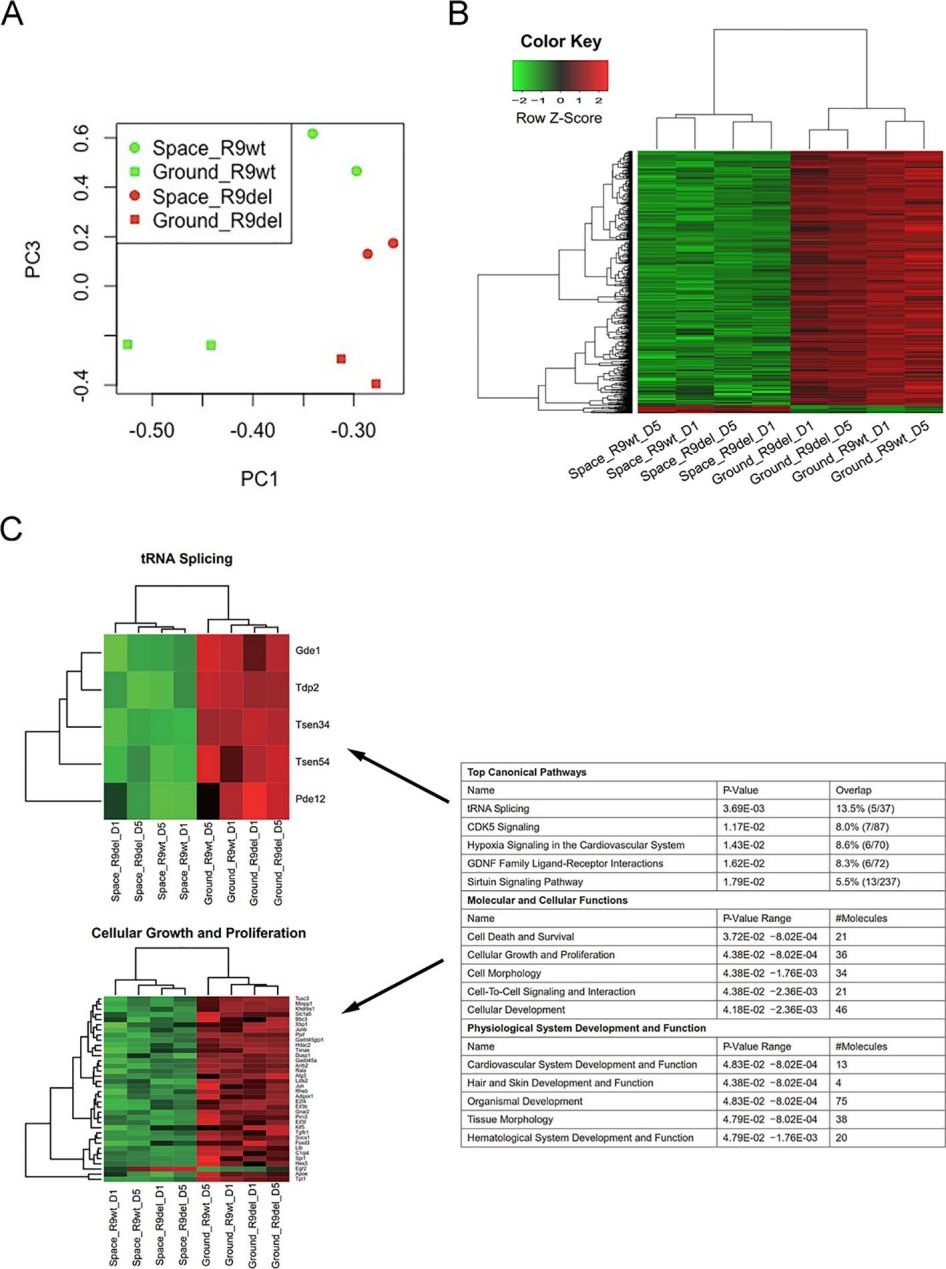
Identification of the DEGs between the space group and the ground group. **(A)** PCA analysis of gene expression across the eight samples was performed to evaluate the variance among samples. Component 1 (PC1) and Component 3 (PC3) are displayed in the dot plot as the *x*- and *y*-axes, respectively. Each point represents a sample. The squares indicate the samples cultured on the ground, while the circles indicate the samples cultured in space. The green dots represent the wild-type mESC (R9wt) samples, while the red dots represent the *Rad9* *^−/−^* mESC (R9del) samples. **(B)** Hierarchical clustering of the expression of the DEGs between the space group and the ground group. **(C)** Enriched functions of the DEGs between the space group and the ground group. The top five enriched functions for the “top canonical pathway,” “molecular and cellular functions,” and “physiological system development and function” categories are listed on the right. All the heat maps for the expression of the genes involved in these 15 functions in both the space group and the ground group can be found in the supplementary data (**Figure S6**). The heat maps for “tRNA splicing” (top) and “cellular growth and proliferation” (bottom) are shown on the left as examples.

As shown in the first four rows of **Table 3**, we identified four sets of DEGs reflecting the effects of spaceflight on the gene expression profiles of mESCs with different genotypes and culture durations. These DEGs were not only influenced by spaceflight but also by Rad9 gene expression and culture duration. To acquire valuable information on the effects of spaceflight on gene expression profiles in mESCs independent of genotype and culture duration, we used two analytical methods. First, we investigated the overlapping genes among these four sets of DEGs. As shown in **Figure S4**, only 1 gene was upregulated and 126 genes were downregulated in both wild-type mESCs and *mRad9* ^−/−^ mESCs subjected to both 1 day of culture and 5 days of culture in space. The limited overlap of the four sets of DEGs made it difficult to analyse the alterations caused by spaceflight independent of both *Rad9* gene expression and culture duration. Second, we applied a group comparison approach. We observed that many DEGs identified by single-sample comparison showed similar expression patterns among space samples or among ground samples but not between space and ground samples (**Figure S5**). These similar patterns were confirmed by the clear overlap among the GO terms for the four sets of DEGs (**Table S1**). These results suggested that we could also explore spaceflight influences by comparing the four space samples with the four ground samples. We thus assigned all eight samples to two groups, the space group and the ground group, each with four replicates, and identified DEGs. Principal component analysis (PCA) indicated that the space group and the ground group differed clearly in Component 3 (PC3; **Figure 4A**), which further supports our strategy of comparing the space samples with the ground samples collectively. With a threshold of fold change ≥ 2 and *p* < 0.05, we identified 596 DEGs (19 upregulated and 577 downregulated) between the space group and the ground group (**Figure 4B**). We further performed a functional analysis of these 596 DEGs. As shown in **Figure 4C**, spaceflight influenced a wide range of cellular functions in mESCs. The top five affected canonical pathways were “tRNA splicing,” “CDK5 signaling,” “hypoxia signaling in the cardiovascular system,” “GDNF family ligand-receptor interactions,” and “sirtuin signaling pathway” (**Figure 4C**).

**Figure 5:**
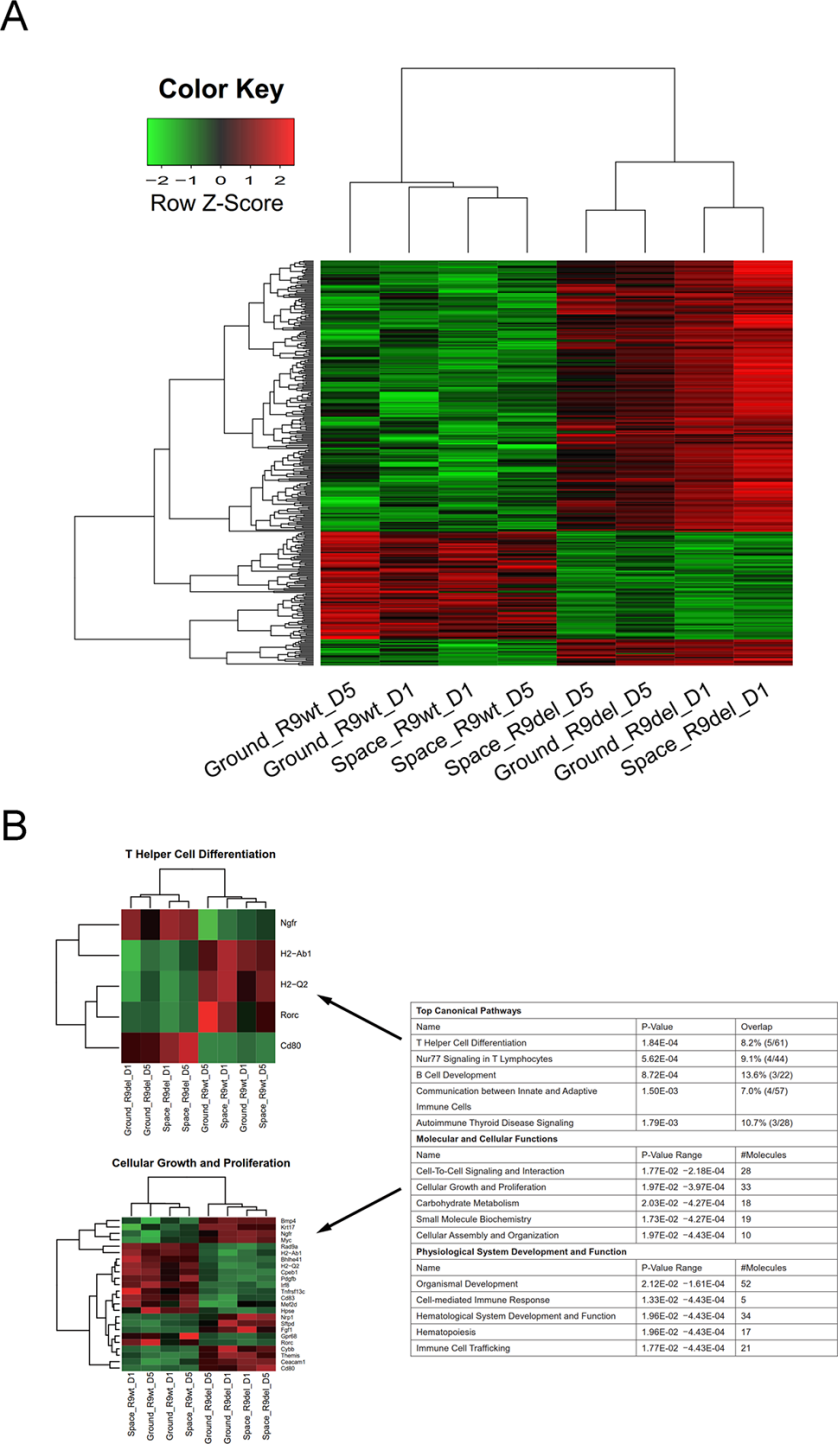
Identification of the DEGs between the R9del group and the R9wt group. **(A)** Hierarchical clustering of the expression of the DEGs between the R9del group and the R9wt group. **(B)** Enriched functions of the DEGs between the R9del group and the R9wt group. The top five enriched functions for the “top canonical pathway,” “molecular and cellular functions,” and “physiological system development and function” categories are listed on the right. All the heat maps for the expression of the genes involved in these 15 functions in both the space group and the ground group can be found in the supplementary data (**Figure S7**). The heat maps for “T helper cell differentiation” (top) and “cellular growth and proliferation” (bottom) are shown on the left as examples. R9wt, wild-type mESCs; R9del, *Rad9* *^−/−^* mESCs.

We also classified the eight samples into two groups by genotype, forming the R9wt group and the R9del group. We identified 238 upregulated genes and 86 downregulated genes upon comparing the R9del group to the R9wt group (**Figure 5A**). The results of the functional analysis of these DEGs are shown in **Figure 5B**. The top five canonical pathways affected by *Rad9* deletion were “T helper cell differentiation,” “Nur77 signaling in T lymphocytes,” “B cell development,” “communication between innate and adaptive immune cells,” and “autoimmune thyroid disease signaling” (**Figure 5B**).

Of note, spaceflight mainly affected organ development and influenced a wide range of cellular functions in mESCs, while *Rad9* deletion mainly affected the development and function of the hematological system, especially the development, differentiation, and function of immune cells. “Cellular growth and proliferation,” “cell-to-cell signalling and interaction,” “organismal development,” and “hematological system development and function” were the only four categories affected by both spaceflight and *Rad9* deletion (**Figures 4C** and **5B**). Even in these four pathways/functions, there were no overlapping genes (**Figures S6 and S7**). Detailed analyses showed that almost all the genes influenced by spaceflight were downregulated, while the genes altered by *Rad9* deletion were neither predominantly downregulated nor upregulated (**Figures S6 and S7**). Therefore, the overall effects of spaceflight and *Rad9* gene deletion on gene expression in mESCs were quite distinct from each other.

### Comparison of the Effects of Spaceflight and *Rad9* Gene Deletion on Genes Encoding Free Radical-Generating Factors, Antioxidants and DNA Repair Factors in mESCs

One objective of our research was to investigate how spaceflight influences the expression of genes critical for inducing, preventing or repairing genomic DNA lesions in mouse mESCs. DNA repair proteins, free radical-generating factors and antioxidants are critical for genome integrity. We were also curious how spaceflight and DNA repair deficiency interact to affect the gene expression of these three classes of proteins. For this purpose, we sent *Rad9* ^−/−^ mESCs (DNA repair-deficient cells) into space in addition to wild-type cells.

**Figure 6:**
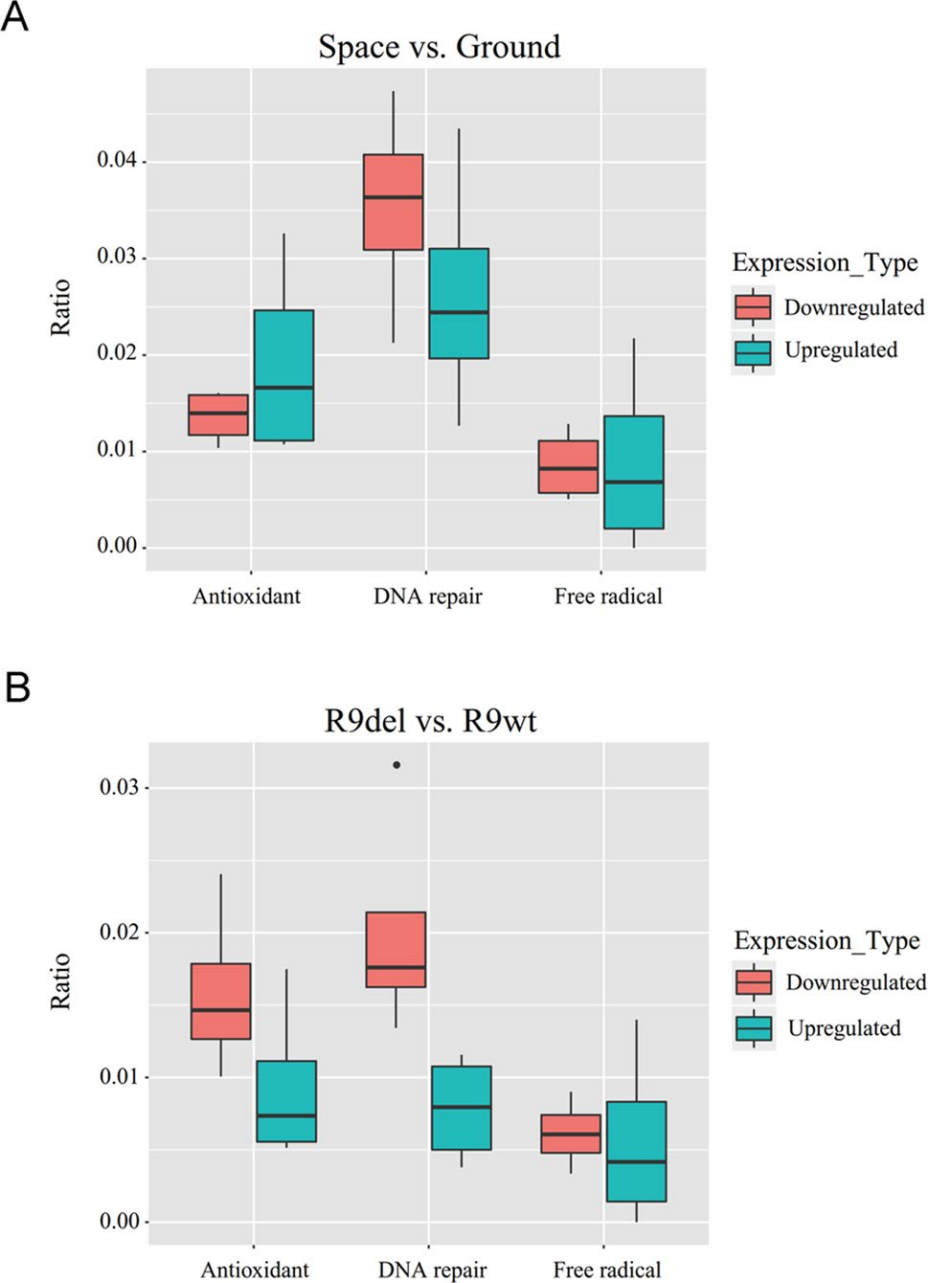
Box plots of the ratios of upregulated and downregulated genes encoding free radical-generating factors, antioxidants and DNA repair factors. **(A)** Median ratios for the genes in the four sets of DEGs reflecting the effects of spaceflight on gene expression. **(B)** Median ratios for the genes in the four sets of DEGs reflecting the effects of *Rad9* deletion on gene expression. Each ratio was calculated by dividing the number of DEGs with the indicated function by the total number of DEGs. The numbers used to calculate the ratios can be found in the supplementary data (**Tables S2 and S3**).

Spaceflight led to more gene downregulation than upregulation in both wild-type and *Rad9* ^−/−^ mESCs (**Table 3**, rows 1 to 4). In contrast, *Rad9* deletion led to more gene upregulation than downregulation (**Table 3**, rows 5 to 8). We then studied whether genes associated with antioxidant activity, free radical production, and DNA repair followed the same global gene expression trends. Spaceflight downregulated more genes than it upregulated, and the downregulated genes included those involved in free radical generation and DNA repair. In contrast, more genes involved in antioxidation were upregulated than downregulated in spaceflight (**Figure 6A**). Although *Rad9* deletion led to more gene upregulation than downregulation in general, *Rad9* deletion led to more downregulation than upregulation among genes involved in antioxidation, free radical generation and DNA repair, indicating that Rad9 plays a unique role in the expression of genes important for genomic integrity (**Figure 6B**). Free radical-generating protein genes and DNA repair genes have opposite functions with regards to genomic DNA integrity. Whether similar trends in the expression of these two types of genes can offset each other is unclear. The above analyses seem to suggest that both spaceflight and *Rad9* deletion downregulated DNA repair genes, which would be harmful to genome integrity.

### Spaceflight-Induced Changes in Global Gene Expression Were Weakened by *Rad9* Deletion

**Figure 7:**
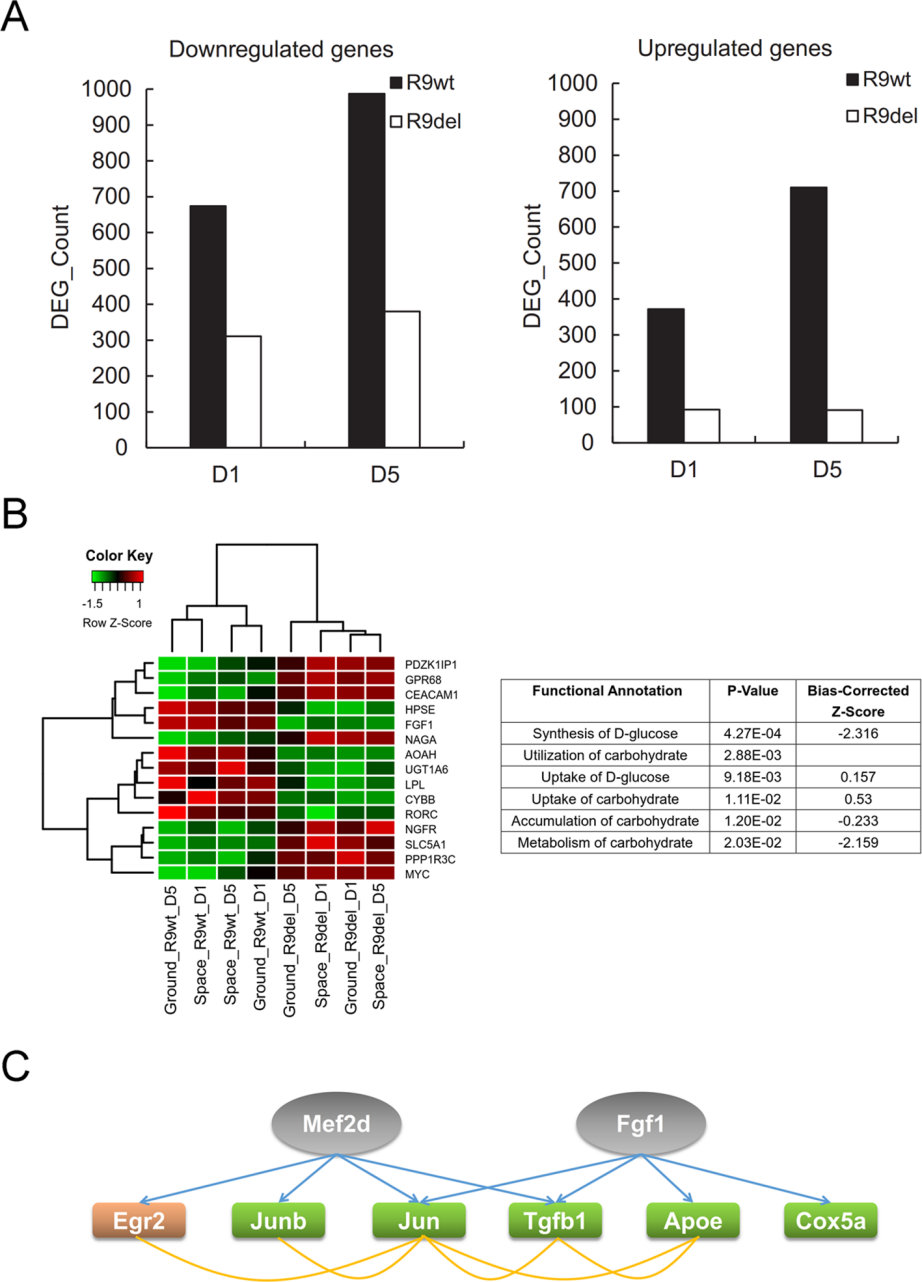
*Rad9* deletion weakens the spaceflight-induced gene expression response in mESCs. **(A)** Comparison of the numbers of DEGs influenced by spaceflight in wild-type (R9wt) mESCs and *Rad9* ^−/−^ (R9del) mESCs after both 1 day (D1) and 5 days (D5) of culture. There were fewer downregulated genes (left) and upregulated genes (right) in *Rad9* ^−/−^ mESCs than in wild-type mESCs. **(B)** Carbohydrate metabolism was affected by *Rad9* deletion according to IPA analysis. Listed are the carbohydrate metabolism-related terms enriched for *Rad9* deletion-influenced DEGs. A negative z-score value indicates downregulation (right). The expression of the DEGs involved in carbohydrate metabolism is presented in the heat map (left). **(C)** Two DEGs (Mef2d and Fgf1, labelled in grey) influenced by *Rad9* deletion can also regulate the expression of six of the DEGs (labelled with orange or green) influenced by spaceflight. Regulating of one gene towards another gene is shown using blue arrow, while associations (either interaction or co-expression or co-localization) between two genes are shown using yellow lines. Orange-labelled genes, genes upregulated by spaceflight; green-labelled genes, genes downregulated by spaceflight; grey-labelled genes, IPA-predicted upstream factors of the orange- and green-labelled genes.

As shown in **Figure 7A**, there were significantly fewer spaceflight-induced upregulated and downregulated DEGs in *Rad9* ^−/−^ mESCs than in wild-type mESCs, indicating that spaceflight-induced changes in global gene expression were weakened by *Rad9* deletion. As mentioned above, we classified the eight samples into two groups by genotype (the R9wt group and R9del group) and identified 238 upregulated genes and 86 downregulated genes in the R9del group compared to the R9wt group (**Figure 5A**). Functional analysis of these DEGs for the “molecular and cellular functions” category indicated that many genes involved in carbohydrate metabolism were clearly altered in the R9del group (**Figure 7B**). This altered carbohydrate metabolism might underlie the weakening of the spaceflight-induced changes in global gene expression in *Rad9* ^−/−^ mESCs. In addition, upstream regulator analysis of spaceflight-altered genes was performed using IPA to get an insight on how these spaceflight-altered genes been affected. Interestingly, two of the DEGs (Mef2d, Fgf1) were predicted as upstream regulator of spaceflight altered genes, implying that downstream genes of Rad9 may help mESCs to react against the spaceflight (**Figure 7C**).

## Discussion

In this study, we investigated the gene expression profiles of mESCs in space and on the ground. We found that spaceflight led to more gene downregulation than upregulation in both wild-type and *Rad9* ^−/−^ mESCs, indicating a suppressive effect of spaceflight on global gene expression (**Table 3**, rows 1 to 4). In contrast, *Rad9* deletion led to more gene upregulation than downregulation (**Table 3**, rows 5 to 8). The DEGs in the comparison between space and ground mESCs were very different from those in the comparison between *Rad9* ^−/−^ and wild-type mESCs (**Figures. S6 and S7**). Despite the differences in global gene expression caused by spaceflight and *Rad9* deletion, more DNA repair genes were downregulated than upregulated under both conditions (**Figure 6**).

More genes were observed to be downregulated than upregulated in both wild-type and *Rad9* ^−/−^ mESCs (**Table 3**, rows 1 to 4). When comparing all four groups of cells in space with the four groups on the ground, the suppressive effect of spaceflight became even more evident (**Figure 4B**). Previous studies on spaceflight-induced changes in the gene expression profiles of other cells or tissues, such as human umbilical vein endothelial cells, fibroblasts and mouse livers (Versari et al., 2013; Zhang et al., 2016; Blaber et al., 2017), did not demonstrate such a dominantly suppressive effect of spaceflight on global gene expression. This might be a unique phenomenon of mESCs experiencing spaceflight. Whether other embryonic stem cells also demonstrate similar suppression of global gene expression during spaceflight needs to be explored. It would be interesting to determine if simulated microgravity also results in similar predominant suppression of global gene expression.

In this study, we identified 596 DEGs that reflected the overall effects of spaceflight on gene expression profiles. Functional analysis of these 596 DEGs showed that the top five affected canonical pathways were “tRNA splicing,” “CDK5 signaling,” “hypoxia signaling in the cardiovascular system,” “GDNF family ligand–receptor interactions,” and “sirtuin signaling pathway”. CDK5 is involved in brain development (Jessberger et al., 2008), hypoxia-induced signalling is involved in the development of the cardiovascular system (Ramírez-Bergeron and Simon, 2010), and GDNF family members play important roles in neurodevelopment (Baloh et al., 2000). Therefore, spaceflight mainly affects the development of organs and influences a wide range of cellular functions (tRNA splicing and the sirtuin signalling pathway) in mESCs. It has previously been reported that after potentially osteogenic murine bone marrow stromal cells are cultured in space for 200 h, most of the modulated genes are those involved in various processes related to neural development, neuron morphogenesis, transmission of nerve impulses and synapses formation (Monticone et al., 2010). Here, we also found that two of the top five canonical pathways affected by spaceflight in mESCs were involved in neurodevelopment. Thus, it will be interesting to further investigate the effects of the space environment on the development and function of the nervous system.

We classified the eight samples into two groups by genotype (the *Rad9* ^−/−^ group and the wild-type group) and identified 324 DEGs. The results of functional analysis of these DEGs showed that *Rad9* deletion mainly affected the development and function of the hematological system, especially the development, differentiation and function of immune cells. The Rad9 protein is well known to play important roles in both cell cycle checkpoint control and DNA repair (Lieberman, 2010). Previously, we generated a conditional knock-out mouse line in which *Rad9* was deleted specifically in B cells and found that Rad9 plays dual roles in generating functional antibodies and in maintaining the integrity of the whole genome in B cells (An et al., 2010). Thus, Rad9 might have broader effects on immune cells than expected through its effects on global gene expression; this possibility deserves further investigation.

Previous research has revealed that Rad9 plays direct roles in several DNA repair pathways by physically interacting with other DNA repair factors. The results of this study showed that Rad9 has a suppressive effect on gene expression in general but that it induces the expression of many DNA repair genes and antioxidant genes. We speculate that when cellular DNA is damaged, it is detrimental for cells to actively express many genes; thus, cells have evolved a mechanism by which Rad9 promotes DNA repair by facilitating DNA repair gene expression while simultaneously inhibiting the expression of genes not involved in DNA repair, thereby reducing vulnerability and minimizing further DNA damage. In general, spaceflight downregulated more genes than it upregulated, and the expression of genes involved in free radical generation and DNA repair followed the general global expression trends. Thus, the impact of spaceflight on the expression of these genes was likely due to non-specific global action. However, more genes involved in antioxidation were upregulated than downregulated in spaceflight (**Figure 6**), indicating a specific effect of spaceflight on the regulation of antioxidation-related genes.

In this study, we sought to determine if spaceflight and *Rad9*-deletion synergistically impaired DNA repair or even genome integrity. However, the gene expression profiles did not give us a simple answer. Both spaceflight and *Rad9* deletion downregulated DNA repair genes, which would be harmful to genome integrity, but their effects on genes enhancing antioxidation and free radical production were complex. Furthermore, free radical-generating and DNA repair genes have opposite effects on genomic DNA integrity. Therefore, it is difficult to determine the overall effects of the combined changes in these genes induced by spaceflight and *Rad9* deletion on genome integrity in mESCs. Due to the limited resources is a weak point of this study, we were unable to send duplicated cell samples to space for this study, and the results from this study should be strengthened or confirmed with similar investigations in the future. Based on the data presented in this study, direct functional studies are needed in space experiments to elucidate the effects of spaceflight on genome integrity.

## Data Availability

The datasets generated for this study can be found in Genome Sequence Archive of Beijing Institute of Genomics, Chinese Academy of Sciences, CRA001384/ http://bigd.big.ac.cn/gsa and in the Gene Expression Omnibus (GEO), under accession numbers GSE134090 (https://www.ncbi.nlm.nih.gov/geo/query/acc.cgi?acc=GSE134090).

## Author Contributions

HH and XF designed research; LA, YF and FR performed research; YL, LA, HH, NH, HQ and XZ analyzed data; HH, LA, YL and YF wrote the paper; YW, CY and YJ contributed new reagents or analytic tools.

## Funding

This work was supported by the National Natural Science Foundation of China (grant number U1738112), the Strategic Priority Research Program of the Chinese Academy of Sciences (grant numbers XDA04020202-13, XDA04020413 and XDA16010209), the National Key Research and Development Program of China (2016YFC0901700) and the National Natural Science Foundation of China (81700097, 81670109).

## Conflict of Interest Statement

The authors declare that the research was conducted in the absence of any commercial or financial relationships that could be construed as a potential conflict of interest.
