# Supplementary material for: The Trends in Global Gene Expression in Mouse Embryonic Stem Cells During Spaceflight
Source: Front Genet. 2019 Sep 6;10:768. doi: 10.3389/fgene.2019.00768 (PMC6743352; doi:10.3389/fgene.2019.00768)
Supplement: Supplementary file 1 [file DataSheet_1.pdf]

Supplementary Material

Figure S1

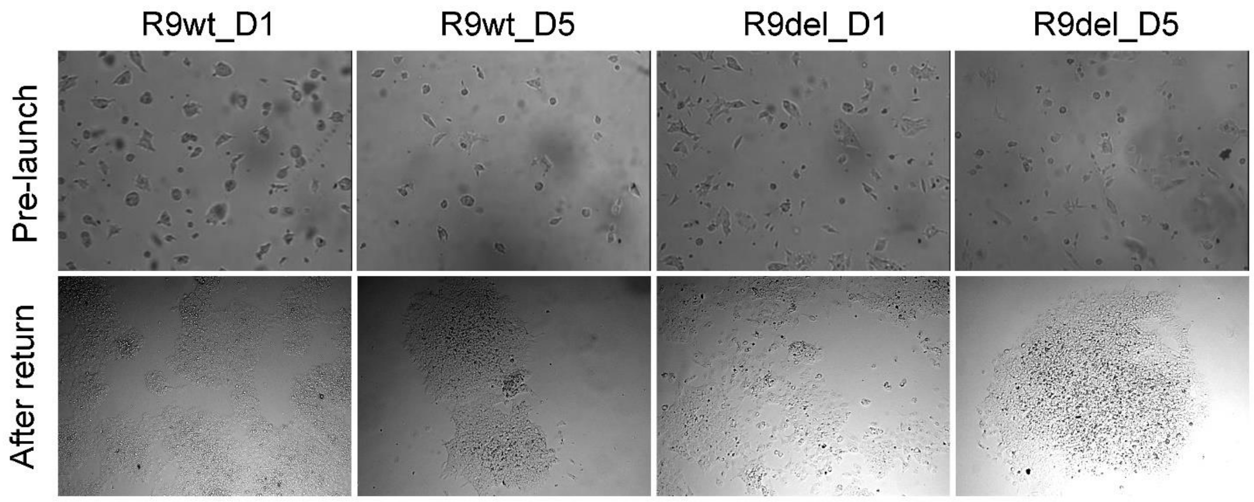

Figure S1 The cells on the cell culture plates before the assembly into the payload (upper) and the fixed cells on the cell culture plates after the return of the satellite (lower). R9wt, wild-type mESCs; R9del, *Rad9*$^{-/-}$ mESCs; D5, mESCs cultured for 5 days in space; D1, mESCs cultured for 1 day in space.

Figure S2

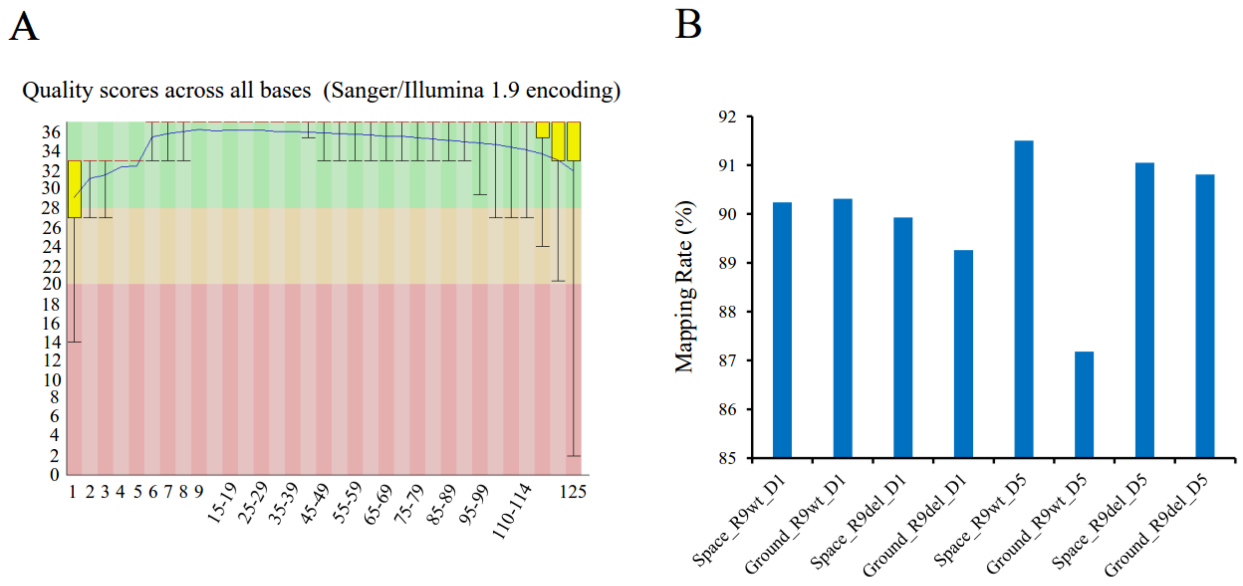

Figure S2 Quality evaluation of RNA-seq data. (A) Representative quality per base as determined with FastQC. (B) Mapping rates of the 8 samples.

Figure S3

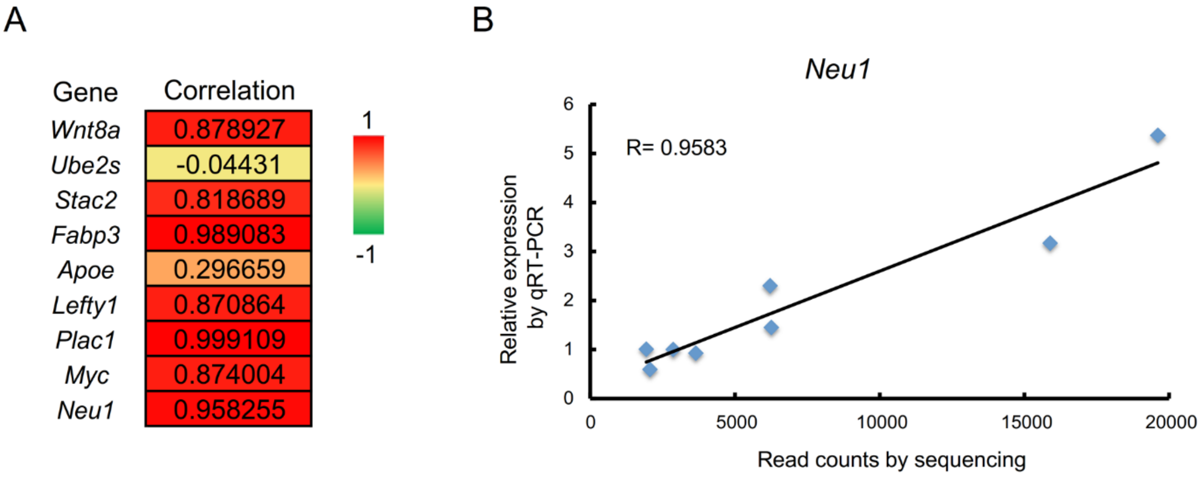

Figure S3 Gene expression comparison between the RNA-seq results and the qRT-PCR results. The expression of 9 randomly selected genes from the RNA-seq data for all 8 samples was further detected using qRT-PCR. For each gene, we compared the results of qRT-PCR and RNA-seq for all 8 samples and calculated the correlation coefficients. A heat map of the expression correlation coefficients for these 9 genes is shown in (A). The value of the correlation coefficient for each gene is labelled on the heat map. The results for the Neu1 gene are shown as an example. The expression levels of the Neu1 gene in the 8 samples detected by qRT-PCR were compared with those detected by RNA-seq, and the results are shown in (B). Each diamond represents a sample, and the expression levels derived from RNA-seq and qRT-PCR are shown on the x axis and y axis, respectively.

Figure S4

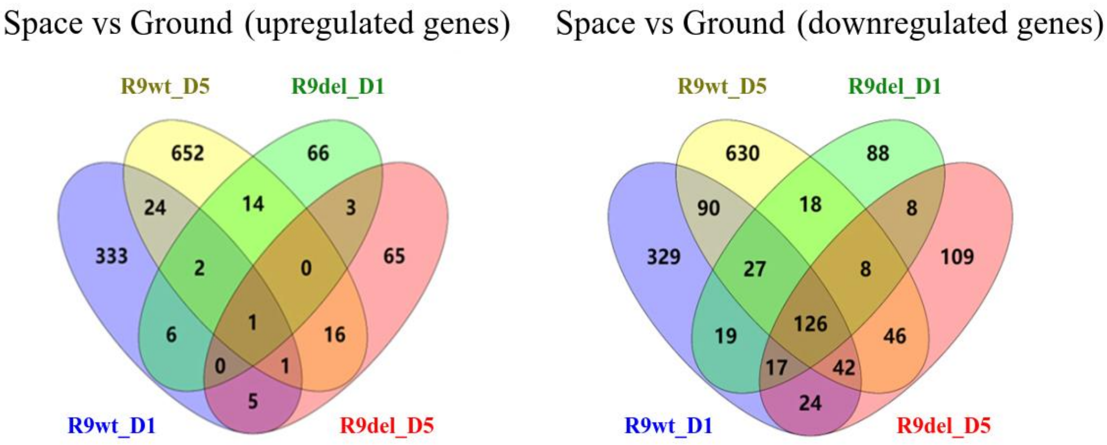

Figure S4 Venn diagrams of the overlapping genes among the four sets of DEGs influenced by spaceflight. (A) Upregulated genes. (B) Downregulated genes.

Figure S5

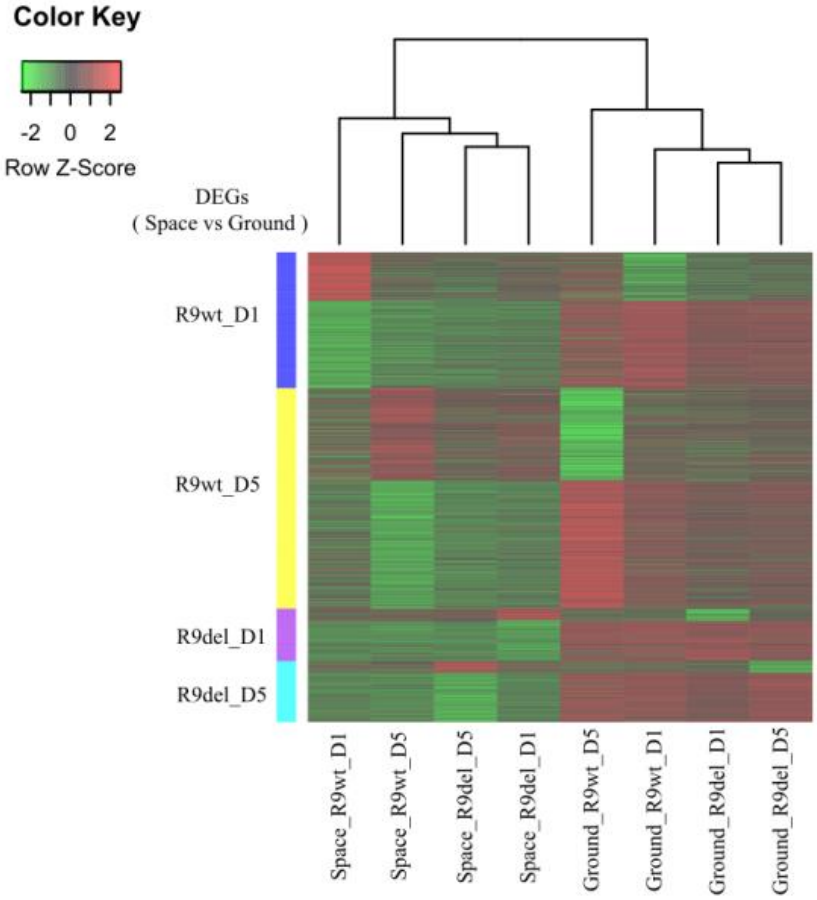

Figure S5 Expression patterns of the four sets of DEGs influenced by spaceflight.

Figure S6

A

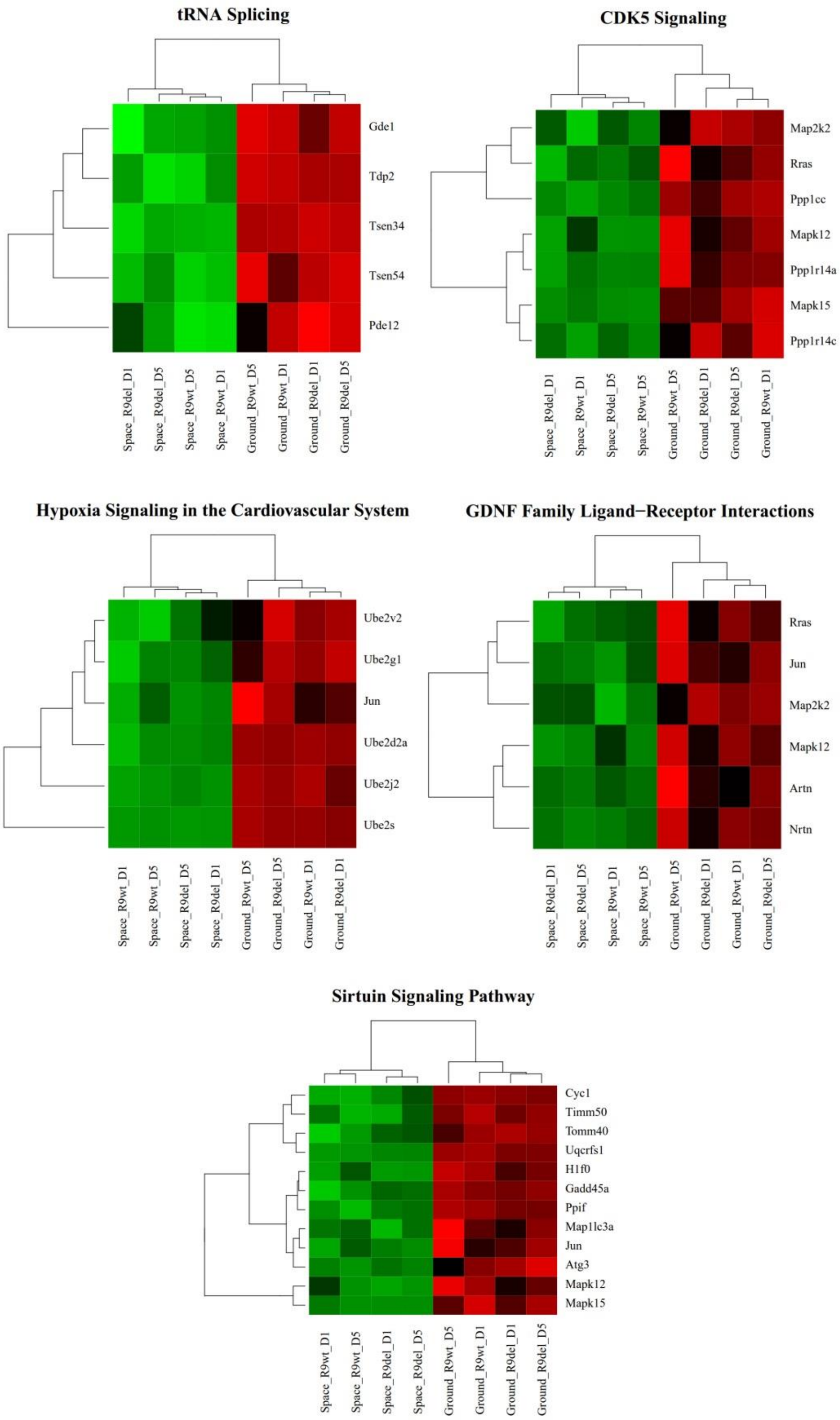
tRNA Splicing
Gde1
Tdp2
Tsen34
Tsen54
Pde12
Space_R9del_D1
Space_R9del_D5
Space_R9wt_D5
Space_R9wt_D1
Ground_R9wt_D5
Ground_R9wt_D1
Ground_R9del_D1
Ground_R9del_D5
CDK5 Signaling
Map2k2
Rras
Ppp1cc
Mapk12
Ppp1r14a
Mapk15
Ppp1r14c
Space_R9del_D1
Space_R9wt_D1
Space_R9del_D5
Space_R9wt_D5
Ground_R9wt_D5
Ground_R9del_D1
Ground_R9del_D5
Ground_R9wt_D1
Hypoxia Signaling in the Cardiovascular System
Ube2v2
Ube2g1
Jun
Ube2d2a
Ube2j2
Ube2s
Space_R9wt_D1
Space_R9wt_D5
Space_R9del_D5
Space_R9del_D1
Ground_R9wt_D5
Ground_R9del_D5
Ground_R9wt_D1
Ground_R9del_D1
GDNF Family Ligand−Receptor Interactions
Rras
Jun
Map2k2
Mapk12
Artn
Nrtn
Space_R9del_D1
Space_R9del_D5
Space_R9wt_D1
Space_R9wt_D5
Ground_R9wt_D5
Ground_R9del_D1
Ground_R9wt_D1
Ground_R9del_D5
Sirtuin Signaling Pathway
Cyc1
Timm50
Tomm40
Uqcrfs1
H1f0
Gadd45a
Ppif
Map1lc3a
Jun
Atg3
Mapk12
Mapk15
Space_R9wt_D1
Space_R9wt_D5
Space_R9del_D1
Space_R9del_D5
Ground_R9wt_D5
Ground_R9wt_D1
Ground_R9del_D1
Ground_R9del_D5

B

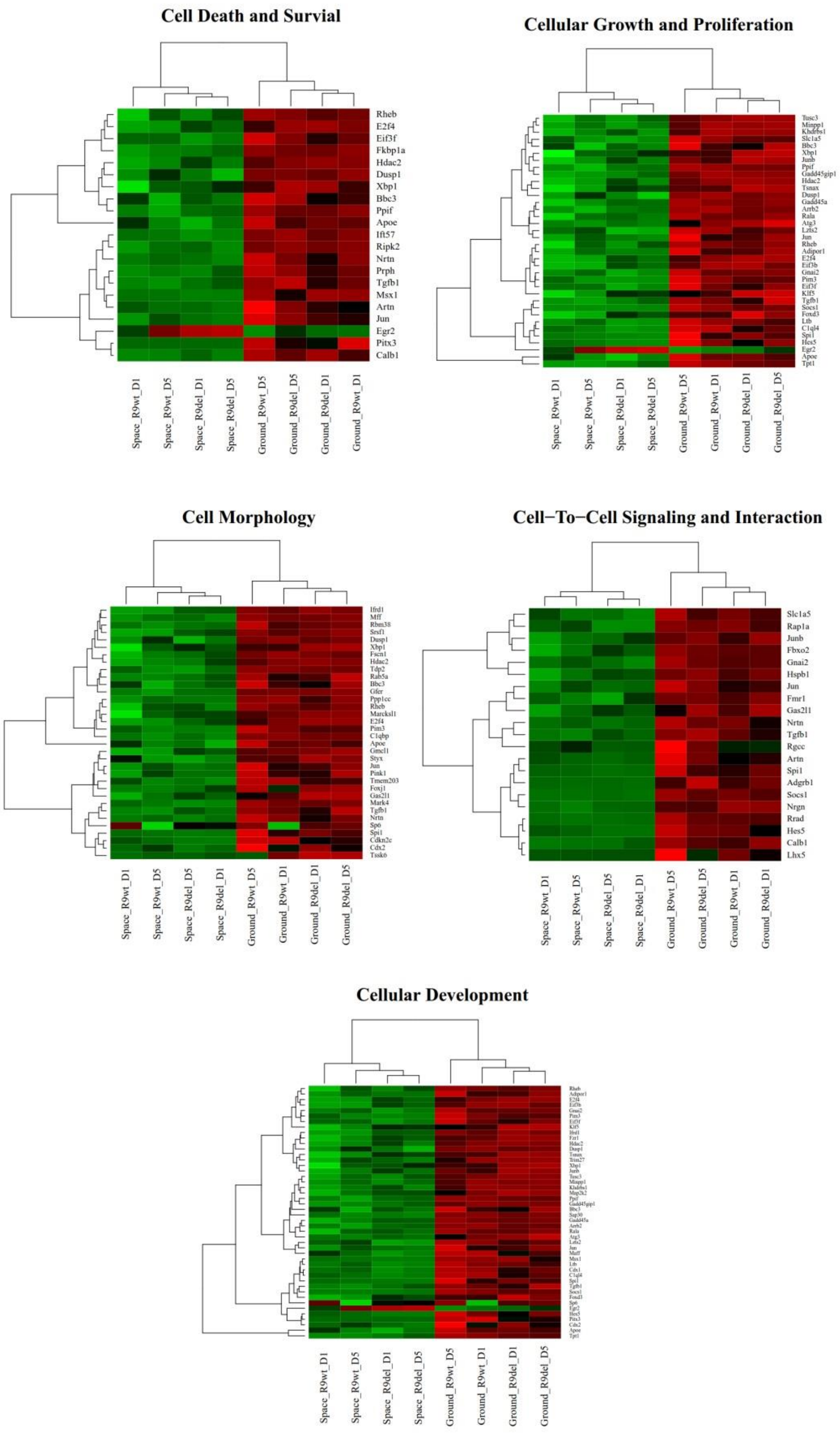
Cell Death and Survial
Rheb
E2f4
Eif3f
Fkbp1a
Hdac2
Dusp1
Xbp1
Bbc3
Ppif
Apoe
Ift57
Ripk2
Nrtn
Prph
Tgfb1
Msx1
Artn
Jun
Egr2
Pitx3
Calb1
Space_R9wt_D1
Space_R9wt_D5
Space_R9del_D1
Space_R9del_D5
Ground_R9wt_D5
Ground_R9del_D5
Ground_R9del_D1
Ground_R9wt_D1
Cellular Growth and Proliferation
Space_R9wt_D1
Space_R9wt_D5
Space_R9del_D1
Space_R9del_D5
Ground_R9wt_D5
Ground_R9wt_D1
Ground_R9del_D1
Ground_R9del_D5
Cell Morphology
Ifrd1
Mff
Rbm38
Srsf1
Dusp1
Xbp1
Fscn1
Hdac2
Tdp2
Rab5a
Bbc3
Gfer
Ppp1cc
Rheb
Marcksl1
E2f4
Pim3
C1qbp
Apoe
Gmcl1
Styx
Jun
Pink1
Tmem203
Foxj1
Gas2l1
Mark4
Tgfb1
Nrtn
Sp6
Spi1
Cdkn2c
Cdx2
Tssk6
Space_R9wt_D1
Space_R9wt_D5
Space_R9del_D5
Space_R9del_D1
Ground_R9wt_D5
Ground_R9wt_D1
Ground_R9del_D1
Ground_R9del_D5
Cell−To−Cell Signaling and Interaction
Slc1a5
Rap1a
Junb
Fbxo2
Gnai2
Hspb1
Jun
Fmr1
Gas2l1
Nrtn
Tgfb1
Rgcc
Artn
Spi1
Adgrb1
Socs1
Nrgn
Rrad
Hes5
Calb1
Lhx5
Space_R9wt_D1
Space_R9wt_D5
Space_R9del_D5
Space_R9del_D1
Ground_R9wt_D5
Ground_R9del_D5
Ground_R9wt_D1
Ground_R9del_D1
Cellular Development
Space_R9wt_D1
Space_R9wt_D5
Space_R9del_D1
Space_R9del_D5
Ground_R9wt_D5
Ground_R9wt_D1
Ground_R9del_D1
Ground_R9del_D5

C

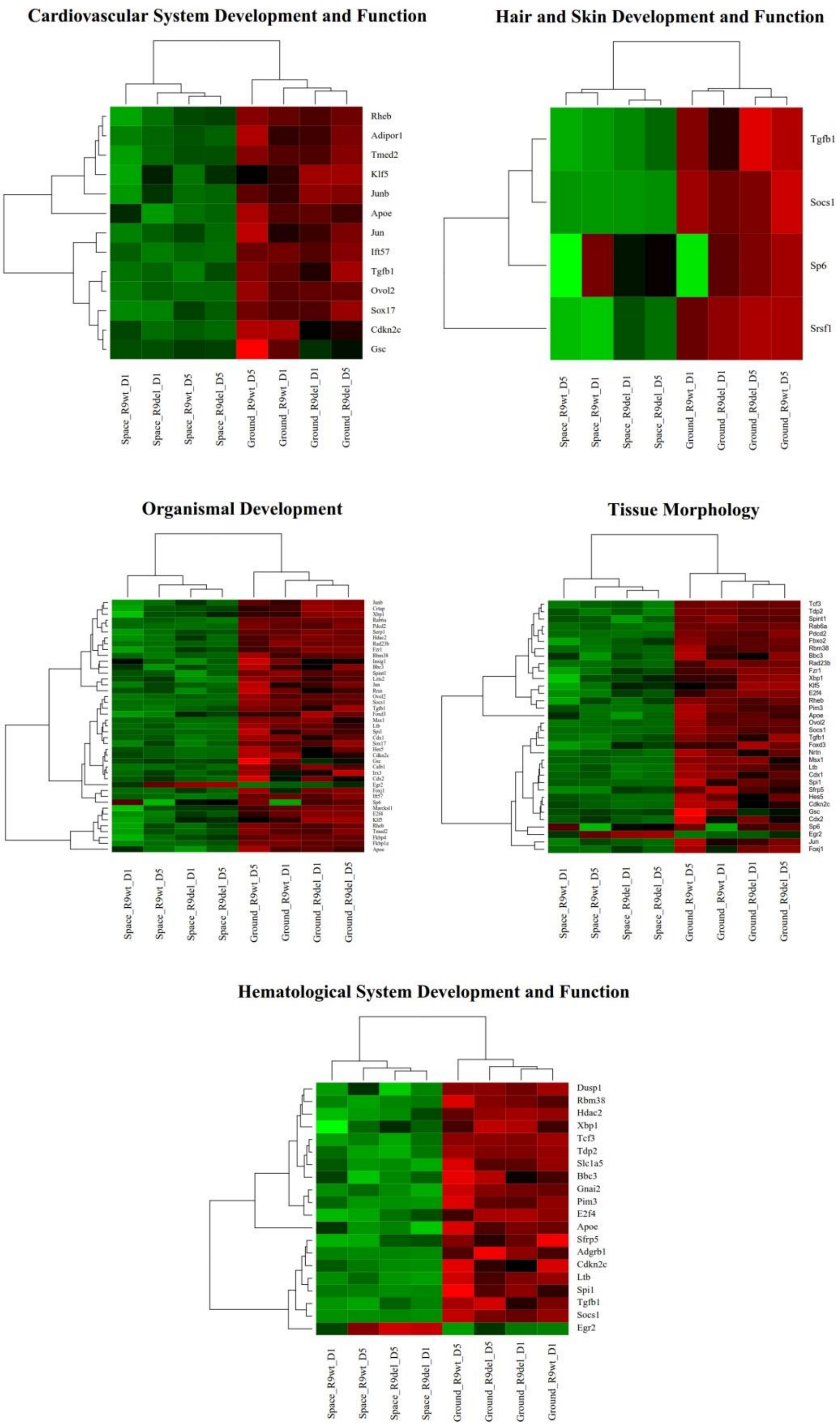
Cardiovascular System Development and Function
Rheb
Adipor1
Tmed2
Klf5
Junb
Apoe
Jun
Ift57
Tgfb1
Ovol2
Sox17
Cdkn2c
Gsc
Space_R9wt_D1
Space_R9del_D1
Space_R9wt_D5
Space_R9del_D5
Ground_R9wt_D5
Ground_R9wt_D1
Ground_R9del_D1
Ground_R9del_D5
Hair and Skin Development and Function
Tgfb1
Socs1
Sp6
Srsf1
Space_R9wt_D5
Space_R9wt_D1
Space_R9del_D1
Space_R9del_D5
Ground_R9wt_D1
Ground_R9del_D1
Ground_R9del_D5
Ground_R9wt_D5
Organismal Development
Space_R9wt_D1
Space_R9wt_D5
Space_R9del_D1
Space_R9del_D5
Ground_R9wt_D5
Ground_R9wt_D1
Ground_R9del_D1
Ground_R9del_D5
Tissue Morphology
Space_R9wt_D1
Space_R9wt_D5
Space_R9del_D1
Space_R9del_D5
Ground_R9wt_D5
Ground_R9wt_D1
Ground_R9del_D1
Ground_R9del_D5
Hematological System Development and Function
Dusp1
Rbm38
Hdac2
Xbp1
Tcf3
Tdp2
Slc1a5
Bbc3
Gnai2
Pim3
E2f4
Apoe
Sfrp5
Adgrb1
Cdkn2c
Ltb
Spi1
Tgfb1
Socs1
Egr2
Space_R9wt_D1
Space_R9wt_D5
Space_R9del_D5
Space_R9del_D1
Ground_R9wt_D5
Ground_R9del_D5
Ground_R9del_D1
Ground_R9wt_D1

Figure S6 Heat maps for the DEGs with the top 5 enriched functions in the “top canonical pathway” (A), “molecular and cellular functions” (B) and “physiological system development and function” (C) categories between the space group and the ground group.

Figure S7

A

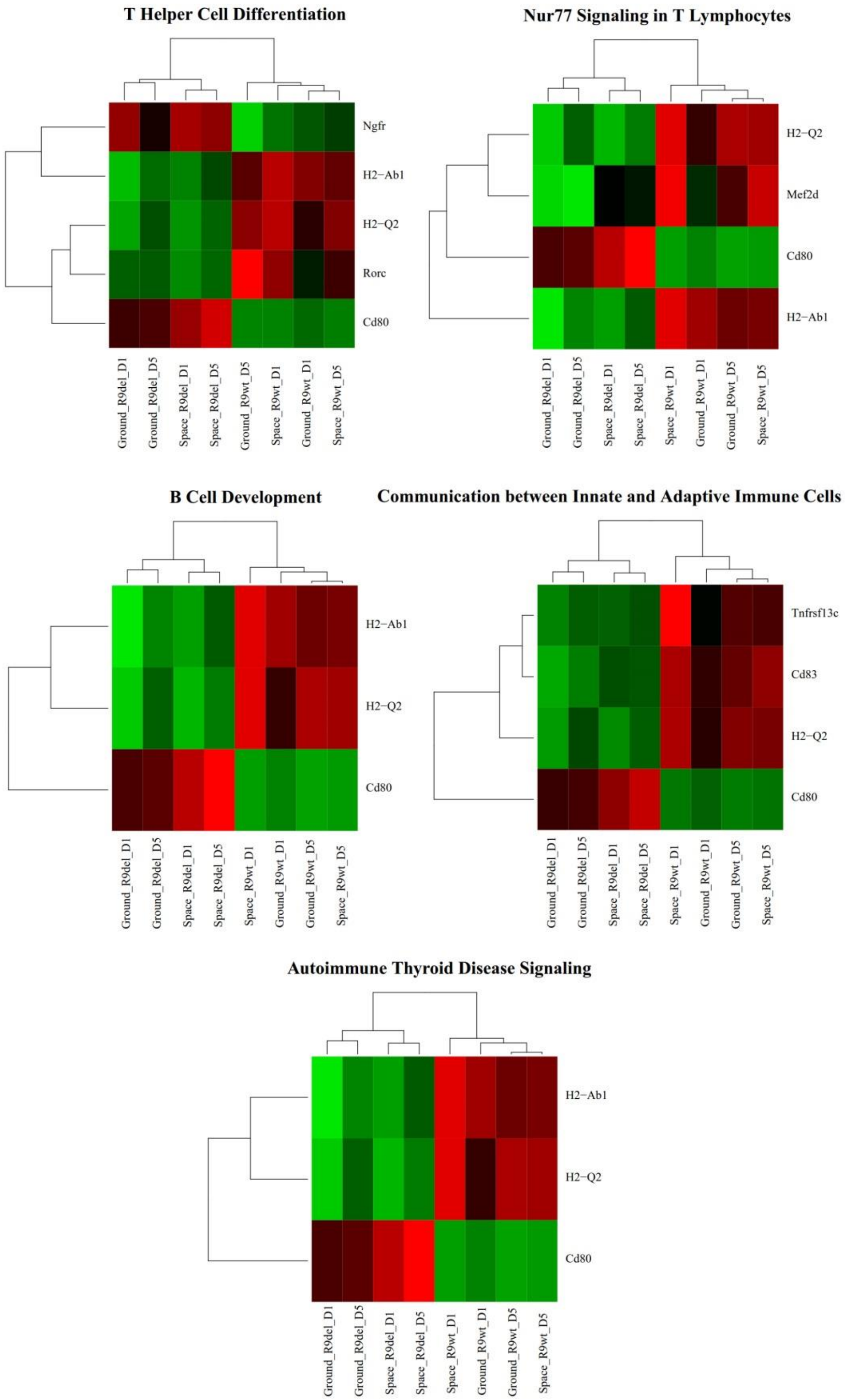
T Helper Cell Differentiation
Ngfr
H2−Ab1
H2−Q2
Rorc
Cd80
Ground_R9del_D1
Ground_R9del_D5
Space_R9del_D1
Space_R9del_D5
Ground_R9wt_D5
Space_R9wt_D1
Ground_R9wt_D1
Space_R9wt_D5
Nur77 Signaling in T Lymphocytes
H2−Q2
Mef2d
Cd80
H2−Ab1
Ground_R9del_D1
Ground_R9del_D5
Space_R9del_D1
Space_R9del_D5
Space_R9wt_D1
Ground_R9wt_D1
Ground_R9wt_D5
Space_R9wt_D5
B Cell Development
H2−Ab1
H2−Q2
Cd80
Ground_R9del_D1
Ground_R9del_D5
Space_R9del_D1
Space_R9del_D5
Space_R9wt_D1
Ground_R9wt_D1
Ground_R9wt_D5
Space_R9wt_D5
Communication between Innate and Adaptive Immune Cells
Tnfrsf13c
Cd83
H2−Q2
Cd80
Ground_R9del_D1
Ground_R9del_D5
Space_R9del_D1
Space_R9del_D5
Space_R9wt_D1
Ground_R9wt_D1
Ground_R9wt_D5
Space_R9wt_D5
Autoimmune Thyroid Disease Signaling
H2−Ab1
H2−Q2
Cd80
Ground_R9del_D1
Ground_R9del_D5
Space_R9del_D1
Space_R9del_D5
Space_R9wt_D1
Ground_R9wt_D1
Ground_R9wt_D5
Space_R9wt_D5

B

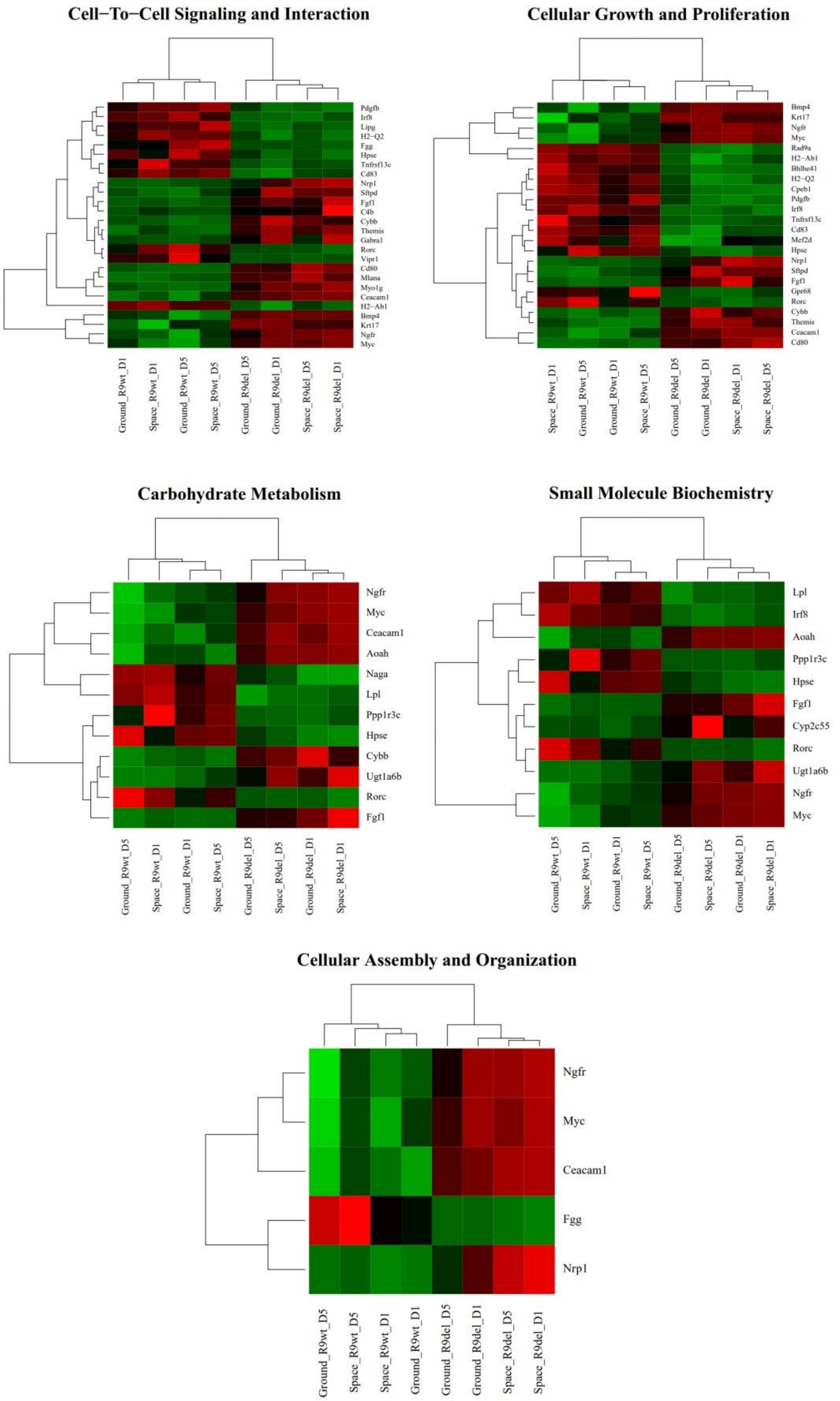
Cell−To−Cell Signaling and Interaction
Pdgfb
Irf8
Lipg
H2−Q2
Fgg
Hpse
Tnfrsf13c
Cd83
Nrp1
Sftpd
Fgf1
C4b
Cybb
Themis
Gabra1
Rorc
Vipr1
Cd80
Mlana
Myo1g
Ceacam1
H2−Ab1
Bmp4
Krt17
Ngfr
Myc
Ground_R9wt_D1
Space_R9wt_D1
Ground_R9wt_D5
Space_R9wt_D5
Ground_R9del_D5
Ground_R9del_D1
Space_R9del_D5
Space_R9del_D1
Cellular Growth and Proliferation
Bmp4
Krt17
Ngfr
Myc
Rad9a
H2−Ab1
Bhlhe41
H2−Q2
Cpeb1
Pdgfb
Irf8
Tnfrsf13c
Cd83
Mef2d
Hpse
Nrp1
Sftpd
Fgf1
Gpr68
Rorc
Cybb
Themis
Ceacam1
Cd80
Space_R9wt_D1
Ground_R9wt_D5
Ground_R9wt_D1
Space_R9wt_D5
Ground_R9del_D5
Ground_R9del_D1
Space_R9del_D1
Space_R9del_D5
Carbohydrate Metabolism
Ngfr
Myc
Ceacam1
Aoah
Naga
Lpl
Ppp1r3c
Hpse
Cybb
Ugt1a6b
Rorc
Fgf1
Ground_R9wt_D5
Space_R9wt_D1
Ground_R9wt_D1
Space_R9wt_D5
Ground_R9del_D5
Space_R9del_D5
Ground_R9del_D1
Space_R9del_D1
Small Molecule Biochemistry
Lpl
Irf8
Aoah
Ppp1r3c
Hpse
Fgf1
Cyp2c55
Rorc
Ugt1a6b
Ngfr
Myc
Ground_R9wt_D5
Space_R9wt_D1
Ground_R9wt_D1
Space_R9wt_D5
Ground_R9del_D5
Space_R9del_D5
Ground_R9del_D1
Space_R9del_D1
Cellular Assembly and Organization
Ngfr
Myc
Ceacam1
Fgg
Nrp1
Ground_R9wt_D5
Space_R9wt_D5
Space_R9wt_D1
Ground_R9wt_D1
Ground_R9del_D5
Ground_R9del_D1
Space_R9del_D5
Space_R9del_D1

C

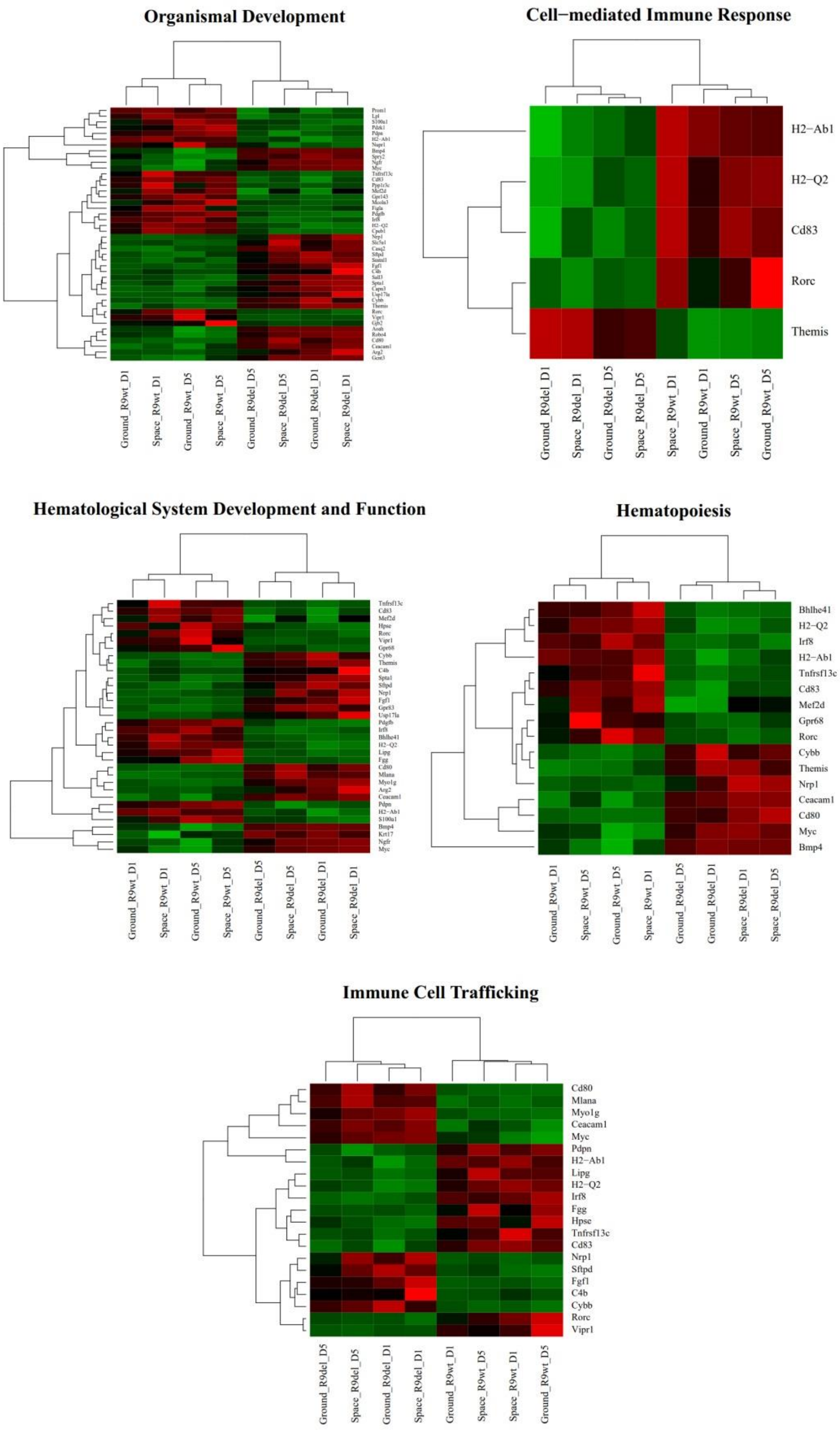
Organismal Development
Ground_R9wt_D1
Space_R9wt_D1
Ground_R9wt_D5
Space_R9wt_D5
Ground_R9del_D5
Space_R9del_D5
Ground_R9del_D1
Space_R9del_D1
Cell−mediated Immune Response
H2−Ab1
H2−Q2
Cd83
Rorc
Themis
Ground_R9del_D1
Space_R9del_D1
Ground_R9del_D5
Space_R9del_D5
Space_R9wt_D1
Ground_R9wt_D1
Space_R9wt_D5
Ground_R9wt_D5
Hematological System Development and Function
Ground_R9wt_D1
Space_R9wt_D1
Ground_R9wt_D5
Space_R9wt_D5
Ground_R9del_D5
Space_R9del_D5
Ground_R9del_D1
Space_R9del_D1
Hematopoiesis
Bhlhe41
H2−Q2
Irf8
H2−Ab1
Tnfrsf13c
Cd83
Mef2d
Gpr68
Rorc
Cybb
Themis
Nrp1
Ceacam1
Cd80
Myc
Bmp4
Ground_R9wt_D1
Space_R9wt_D5
Ground_R9wt_D5
Space_R9wt_D1
Ground_R9del_D5
Ground_R9del_D1
Space_R9del_D1
Space_R9del_D5
Immune Cell Trafficking
Cd80
Mlana
Myo1g
Ceacam1
Myc
Pdpn
H2−Ab1
Lipg
H2−Q2
Irf8
Fgg
Hpse
Tnfrsf13c
Cd83
Nrp1
Sftpd
Fgf1
C4b
Cybb
Rorc
Vipr1
Ground_R9del_D5
Space_R9del_D5
Ground_R9del_D1
Space_R9del_D1
Ground_R9wt_D1
Space_R9wt_D5
Space_R9wt_D1
Ground_R9wt_D5

Figure S7 Heat maps for the DEGs with the top 5 enriched functions in the “top canonical pathway” (A), “molecular and cellular functions” (B) and “physiological system development and function” (C) categories between the *Rad9*$^{-/-}$ group and the wild-type group.

Supplementary Table S1. Top enriched GO functions

Space R9wt D1/Ground R9wt D1

**Description**	**qvalue**
regionalization	0.0001058
transcriptional activator activity, RNA polymerase II transcription regulatory region sequence-specific binding	0.000573935
pattern specification process	0.000823056
somitogenesis	0.001470888
somite development	0.00200668
segmentation	0.00316559
transcription factor activity, RNA polymerase II distal enhancer sequence-specific binding	0.003331251
RNA polymerase II core promoter proximal region sequence-specific DNA binding	0.003495881
core promoter proximal region sequence-specific DNA binding	0.003495881
core promoter proximal region DNA binding	0.003495881

Space R9wt D5/Ground R9wt D5

**Description**	**qvalue**
transcriptional activator activity, RNA polymerase II transcription regulatory region sequence-specific binding	5.22E-10
cell fate commitment	2.83E-09
mesenchyme development	2.83E-09
pattern specification process	4.89E-09
heart morphogenesis	4.98E-08
muscle organ development	2.16E-07
embryonic organ morphogenesis	3.58E-07
regionalization	6.71E-07
embryonic organ development	6.71E-07
tube morphogenesis	1.27E-06

Space R9del D1/Ground R9del D1

**Description**	**qvalue**

regionalization	0.000744028
cation channel complex	0.000905146
pattern specification process	0.001166998
nephron tubule formation	0.007174617
acylglycerol homeostasis	0.008679162
triglyceride homeostasis	0.008679162
transmembrane transporter complex	0.010505163
ion channel complex	0.010505163
transporter complex	0.010505163
transcriptional activator activity, RNA polymerase II transcription regulatory region sequence-specific binding	0.012686076

Space R9del D5/Ground R9del D5

**Description**	**qvalue**
transcription factor activity, RNA polymerase II core promoter proximal region sequence-specific binding	4.35E-05
core promoter proximal region DNA binding	6.52E-05
transcriptional activator activity, RNA polymerase II transcription regulatory region sequence-specific binding	6.52E-05
core promoter proximal region sequence-specific DNA binding	8.42E-05
RNA polymerase II core promoter proximal region sequence-specific DNA binding	0.000134482
transcriptional activator activity, RNA polymerase II core promoter proximal region sequence-specific binding	0.000229727
regionalization	0.001216296
pattern specification process	0.002618643
transcriptional repressor activity, RNA polymerase II transcription regulatory region sequence-specific binding	0.00581399
regulation of Wnt signaling pathway	0.006545893

R9del Space D1/R9wt Space D1

**Description**	**qvalue**
blood circulation	4.41E-08
circulatory system process	4.41E-08
muscle contraction	5.54E-08
regulation of system process	2.37E-07
regulation of blood circulation	2.24E-06
substrate-specific channel activity	1.33E-05
ion channel activity	1.33E-05
transcriptional activator activity, RNA polymerase II transcription	1.33E-05

regulatory region sequence-specific binding	
channel activity	1.33E-05
passive transmembrane transporter activity	1.33E-05

R9del Space D5/R9wt Space D5

Description	qvalue
proteinaceous extracellular matrix	4.40E-12
extracellular matrix	3.10E-10
ion channel activity	3.37E-10
substrate-specific channel activity	3.37E-10
gated channel activity	5.68E-10
channel activity	5.68E-10
passive transmembrane transporter activity	5.68E-10
regulation of hormone levels	1.53E-08
growth factor activity	4.50E-08
ion channel complex	8.39E-08

R9del Ground D1/R9wt Ground D1

Description	qvalue
regulation of system process	2.67E-07
proteinaceous extracellular matrix	3.38E-06
muscle contraction	7.08E-06
ion channel complex	1.13E-05
regulation of ion transmembrane transport	1.30E-05
muscle system process	1.50E-05
phenol-containing compound metabolic process	2.70E-05
regulation of transmembrane transport	3.07E-05
transmembrane transporter complex	3.82E-05
extracellular matrix	3.82E-05

R9del Ground D5/R9wt Ground D5

Description	qvalue
substrate-specific channel activity	2.09E-09
channel activity	2.09E-09
passive transmembrane transporter activity	2.09E-09
ion channel activity	2.09E-09
regulation of system process	9.64E-09
cellular calcium ion homeostasis	1.82E-06

calcium ion homeostasis	1.82E-06
pattern specification process	3.29E-06
cellular divalent inorganic cation homeostasis	3.29E-06
transcriptional activator activity, RNA polymerase II transcription regulatory region sequence-specific binding	3.65E-06

D5 Space R9wt /D1 Space R9wt

**Description**	**qvalue**
angiogenesis	2.74E-12
extracellular matrix	2.38E-11
proteinaceous extracellular matrix	4.79E-09
cell fate commitment	9.74E-09
regulation of body fluid levels	1.18E-07
wound healing	2.91E-07
anion transport	3.22E-07
regulation of hormone levels	3.69E-07
embryonic organ development	5.86E-07
regulation of system process	5.86E-07

D5 Space R9del /D1 Space R9del

**Description**	**qvalue**
regulation of hormone levels	0.000415234
digestion	0.000415234
lipid digestion	0.000415234
channel activity	0.000725287
passive transmembrane transporter activity	0.000725287
substrate-specific channel activity	0.000725287
ion channel activity	0.000725287
brush border membrane	0.001639238
cluster of actin-based cell projections	0.001639238
transcriptional activator activity, RNA polymerase II transcription regulatory region sequence-specific binding	0.002374184

D5 Ground R9wt /D1 Ground R9wt

**Description**	**qvalue**
regulation of system process	1.15E-12
blood circulation	4.25E-09

circulatory system process	4.93E-09
transcriptional activator activity, RNA polymerase II transcription regulatory region sequence-specific binding	2.64E-08
pattern specification process	5.61E-08
divalent inorganic cation homeostasis	6.92E-08
calcium ion homeostasis	6.92E-08
cellular divalent inorganic cation homeostasis	8.15E-08
regulation of blood circulation	8.25E-08
cellular calcium ion homeostasis	8.41E-08

D5 Ground R9del /D1 Ground R9del

**Description**	**qvalue**
cellular calcium ion homeostasis	0.001126988
calcium ion homeostasis	0.001126988
cellular divalent inorganic cation homeostasis	0.001333137
divalent inorganic cation homeostasis	0.001684478
regulation of system process	0.028753071
neuropeptide signaling pathway	0.028753071
regulation of synapse organization	0.028753071
regulation of synapse assembly	0.028753071
synapse organization	0.028753071
regulation of cytosolic calcium ion concentration	0.028753071

Supplementary Table S2. The ratios of the DEGs caused by spaceflight with indicated functions

Anti-oxidant	Fixed conditions	Number of upregulated genes (name of upregulated genes)	Number of total upregulated genes	Ratio	Number of downregulated genes (name of downregulated genes)	Number of total downregulated genes	Ratio
	R9wt_D1	4 (Tpo, Ltc4s, Atp7a, Hp)	372	0.010753	7 (Lpo, Apoe, Rgn, Srxn1, Gpx2, Aaed1, Gpx1)	674	0.010386
	R9wt_D5	8 (Sod3, Lpo, Mgst2, Txndc2, Mt4, Meltf, Ptgs2, Ubiad1)	710	0.011268	12 (Hba-a1, Hba-a2, Cp, Iyd, Apoe, Gpx6, Ltf, Trp53inp1, Nxnl2, Epx, Gstt2, Aaed1)	987	0.012158
	R9del_D1	3 (Alb, Mgst2, Txndc2)	92	0.032609	5 (Apoe, Srxn1, Cp, Nxnl1, Aaed1)	311	0.016077
	R9del_D5	2 (Mgst2, Sod3)	91	0.021978	6 (Nxnl1, Apoe, Aaed1, Srxn1, Hba-a1, Nxnl2)	380	0.015789
**Free radical**	**Fixed conditions**	**Number of upregulated genes (name of upregulated genes)**	**Number of total upregulated genes**	**Ratio**	**Number of downregulated genes (name of downregulated genes)**	**Number of total downregulated genes**	**Ratio**
	R9wt_D1	1 (Duox2)	372	0.002688	4 (Uqcrfs1, Cyc1, mt-Nd3, Ndufab1)	674	0.005935
	R9wt_D5	0	710	0	5 (Pax2, Uqcrfs1, Cyc1, Duox2, Ndufab1)	987	0.005066
	R9del_D1	2 (Duox1, Duox2)	92	0.021739	4 (Uqcrfs1, Cyc1, mt-Nd3, Ndufab1)	311	0.012862
	R9del_D5	1 (Noxa1)	91	0.010989	4 (Uqcrfs1, Cyc1, Pax2, mt-Nd3)	380	0.010526
**DNA repair**	**Fixed conditions**	**Number of upregulated genes (name of upregulated genes)**	**Number of total upregulated genes**	**Ratio**	**Number of downregulated genes (name of downregulated genes)**	**Number of total downregulated genes**	**Ratio**
	R9wt_D1	10 (Per1, Polk, Gadd45g, Trp53tg5, Msh4, Chd9, Msh5, Btg2, Trex1, Polh)	372	0.026882	24 (Trex2, H2afx, Ino80b, Gadd45a, Asf1a, Nudt11, Rfc5, Rad23b, Piwil4, Znhit1, Ung, Tdp2, Setd6, Sfpq, Fance, Nudt19, Hmg20b, Xpa, Dek, Clk2, Cetn2, Ube2v2, Smarcd3, Cry1)	674	0.035608
	R9wt_D5	9 (Zhx1, Mlh3, Ercc6l, Trex1, Dpf3, Fen1, Ino80c, Pml, Dclre1a)	710	0.012676	21 (H2afx, Gadd45a, Setd6, Ino80b, Fance, Tdp2, Gadd45b, Pttg1, Asf1a, Xpa, Ung, Cebpg, Wrnip1, Rad23b, Yy1, Gadd45g, Hmg20b, Polg2, Smarcd3, Ube2v2, Cry1)	987	0.021277
	R9del_D1	4 (Ube2v1, Pttg1ip, Spo11, Fen1)	92	0.043478	12 (H2afx, Ino80b, Asf1a, Cebpg, Gadd45a, Fance, Rad23b, Tdp2, Wrnip1, Ube2v2, Ung, Clk2)	311	0.038585
	R9del_D5	2 (Ikzf1, Pttg1ip)	91	0.021978	18 (Actl6b, Cebpg, Ino80b, Asf1a, H2afx, Setd6, Gadd45a, Fance, Xpa, Dek, Ube2v2, Tdp2, Rad23b, Wrnip1, Clk2, Nbn, Hmg20b, Yy1)	380	0.047368

Supplementary Table S3. The ratios of the DEGs caused by Rad9-deletion with indicated functions

**R9del VS. R9wt**							
**Anti-oxidant**	**Fixed conditions**	**Number of upregulated genes (name of upregulated genes)**	**Number of total upregulated genes**	**Ratio**	**Number of downregulated genes (name of downregulated genes)**	**Number of total downregulated genes**	**Ratio**
	Space_D1	10 (Lpo, Alb, Ltf, Iyd, Gpx3, Cygb, Txndc2, Ptgs2, Gpx2, Nqo1)	1110	0.009009	6 (Gstt2, Gstt1, Nxnl1, Gstm7, Pxdn, Atp7a)	596	0.010067
	Space_D5	5 (Hba-a2, Iyd, Cp, Hba-a1, Ptgs1)	286	0.017483	7 (Gpx5, Nxnl1, Tpo, Ptgs2, Meltf, Gpx8, Nxnl2)	291	0.024055
	Ground_D1	3 (Gpx3, Cp, Tpo)	527	0.005693	3 (Mgst2, Rgn, Mgst1)	190	0.015789
	Ground_D5	4 (Lpo, Txndc2, Mt4, Srxn1)	778	0.005141	6 (Gstt2, Gpx6, Nxnl2, Fam213a, Ltf, Gstt1)	444	0.013514
**Free radical**	**Fixed conditions**	**Number of upregulated genes (name of upregulated genes)**	**Number of total upregulated genes**	**Ratio**	**Number of downregulated genes (name of downregulated genes)**	**Number of total downregulated genes**	**Ratio**
	Space_D1	0	1110	0	2 (Pdgfb, Cyba)	596	0.003356
	Space_D5	4 (Pax2, Cybb, Nox1, Duox1)	286	0.013986	2 (Pdgfb, Nox4)	291	0.006873
	Ground_D1	1 (Cybb)	527	0.001898	1 (Pdgfb)	190	0.005263
	Ground_D5	5 (Nox1, mt-Nd3, mt-Cytb, Cybb, Noxo1)	778	0.006427	4 (Noxa1, Duox2, Cyba, Pdgfb)	444	0.009009
**DNA repair**	**Fixed conditions**	**Number of upregulated genes (name of upregulated genes)**	**Number of total upregulated genes**	**Ratio**	**Number of downregulated genes (name of downregulated genes)**	**Number of total downregulated genes**	**Ratio**
	Space_D1	6 (Actl6b, Spo11, Fancf, Gm9840, Ercc6l, Fen1)	1110	0.005405	8 (Gadd45g, Gadd45b, Ctcfl, Polk, Nudt18, Cebpg, Btg2, Pttg1)	596	0.013423
	Space_D5	3 (Piwil4, Spta1, Trp73)	286	0.01049	5 (Trex1, Gadd45g, Cebpg, Gadd45b, Cul4b)	291	0.017182
	Ground_D1	2 (Spta1, Trex1)	527	0.003795	6 (Spo11, Pttg1ip, Neil2, Rad9a, Fignl1, Smarcd3)	190	0.031579
	Ground_D5	9 (Actl6b, Trp53tg5, Spta1, Gm9840, Zhx1, Fen1, Ercc6l, Fancf, Mlh3)	778	0.011568	8 (Trex2, Ikzf1, Gadd45g, Gadd45b, Pttg1ip, Ccno, Ctcfl, Dmc1)	444	0.018018
